# Monocyte Recruitment to the Dermis and Differentiation to Dendritic Cells Increases the Targets for Dengue Virus Replication

**DOI:** 10.1371/journal.ppat.1004541

**Published:** 2014-12-04

**Authors:** Michael A. Schmid, Eva Harris

**Affiliations:** Division of Infectious Diseases & Vaccinology, School of Public Health, University of California Berkeley, Berkeley, California, United States of America; Purdue University, United States of America

## Abstract

Dengue virus (DENV) causes the most prevalent arthropod-borne viral disease in humans. Although *Aedes* mosquitoes transmit DENV when probing for blood in the skin, no information exists on DENV infection and immune response in the dermis, where the blood vessels are found. DENV suppresses the interferon response, replicates, and causes disease in humans but not wild-type mice. Here, we used mice lacking the interferon-α/β receptor (*Ifnar* ^–/–^), which had normal cell populations in the skin and were susceptible to intradermal DENV infection, to investigate the dynamics of early DENV infection of immune cells in the skin. CD103^+^ classical dendritic cells (cDCs), Ly6C^–^ CD11b^+^ cDCs, and macrophages in the steady-state dermis were initial targets of DENV infection 12-24 hours post-inoculation but then decreased in frequency. We demonstrated recruitment of adoptively-transferred Ly6C^high^ monocytes from wild-type and *Ifnar* ^–/–^ origin to the DENV-infected dermis and differentiation to Ly6C^+^ CD11b^+^ monocyte-derived DCs (moDCs), which became DENV-infected after 48 hours, and were then the major targets for virus replication. Ly6C^high^ monocytes that entered the DENV-infected dermis expressed chemokine receptor CCR2, likely mediating recruitment. Further, we show that ∼100-fold more hematopoietic cells in the dermis were DENV-infected compared to Langerhans cells in the epidermis. Overall, these results identify the dermis as the main site of early DENV replication and show that DENV infection in the skin occurs in two waves: initial infection of resident cDCs and macrophages, followed by infection of monocytes and moDCs that are recruited to the dermis. Our study reveals a novel viral strategy of exploiting monocyte recruitment to increase the number of targets for infection at the site of invasion in the skin and highlights the skin as a potential site for therapeutic action or intradermal vaccination.

## Author Summary

The skin and its immune cells are an important barrier against invading pathogens. Dengue is a major public health problem worldwide, with no specific therapeutic or vaccine available. *Aedes* mosquitoes transmit dengue virus (DENV) to humans via the skin when taking a blood meal. Previous studies have examined DENV infection only in the epidermis, the uppermost layer of the skin, but no information existed about DENV infection in the dermis, the layer below that contains blood vessels. We established a model of DENV infection in the skin of mouse ears, as biopsies from naturally-infected human skin are unavailable. The normal dermis contains classical dendritic cells (DCs) and macrophages, which we found to be the initial targets of DENV infection. Monocytes that circulate in the blood were then recruited to the dermis and differentiated to monocyte-derived DCs, an inflammatory DC subset. These newly-recruited monocytes and monocyte-derived DCs became DENV-infected in a second wave and then were the major targets for DENV replication. Our study identifies how DENV exploits the immune response by infecting cells that are recruited to the skin as part of antiviral defense. These results should help future research to develop new strategies for vaccination and therapeutics against dengue.

## Introduction

The skin is the barrier to the environment and provides a first line of defense against invasion of microbial pathogens. Dendritic cells (DCs) and macrophages (MΦs) serve as immune sentinels in the skin [1]. DCs take up antigen, sense the presence of invading pathogens, and migrate to draining lymph nodes (LNs), where they prime naïve T cells [2]. MΦs are tissue-resident cells that are specialized in phagocytosis and local antigen presentation to effector and memory T cells [3].

Several subsets of DCs have been identified in the steady-state skin. The epidermis contains Langerhans cells (LCs) that self-renew [4]. The dermis of mice contains CD103^+^ classical DCs (cDCs) and CD11b^+^ DCs [5], [6] that are replenished by blood-derived precursors. In other non-lymphoid tissues, CD103^+^ cDCs are derived from pre-cDCs – precursors down-stream of common DC progenitors [7]–[10]. CD11b^+^ DCs are derived from pre-cDCs as well as from monocytes [11], suggesting that CD11b^+^ DCs are heterogeneous and need to be further resolved. Additionally, the entry of pre-cDCs into the steady-state dermis and replenishment of dermal DCs has not been demonstrated.

Inflammation drastically changes the network of immune cells in the skin. Ultraviolet light, chemicals, or herpes simplex virus-1 infection induce the migration of epidermal LCs [4] and dermal DCs [12], [13] to LNs, where they prime CD4^+^ and CD8^+^ T cell responses. Ly6C^high^ monocytes enter the inflamed epidermis to replenish LCs [14] and are recruited to other inflamed tissues, where they differentiate to monocyte-derived DCs (moDCs) [15]. Two studies showed monocyte recruitment and differentiation to moDCs in the inflamed dermis during *Leishmania major* infection [16] and contact hypersensitivity reaction [17]. Yet, many questions remain as to how DCs are replenished in the inflamed dermis and how pathogens overcome the immune response in the skin to establish infection.

The four dengue virus serotypes (DENV1–4) cause the most common arthropod-borne viral disease of humans, with 390 million infections and up to 96 million cases of dengue per year [18]. No specific vaccine or therapeutic exists against dengue. DENV is a *Flavivirus* that contains a positive-strand RNA genome encoding 3 structural (C, prM/M, E) and 7 non-structural proteins [19]. *Aedes aegypti* and *Ae. albopictus* mosquitoes transmit DENV when probing for blood vessels in the dermis [20]. After systemic spread, monocytes, DCs, and MΦs are the main targets for DENV replication [21]–[23]. The few studies that have examined the skin found DENV infection in epidermal LCs [24]–[26]; however, no information exists about DENV infection and the immune response in the dermis, where DENV is most likely transmitted.

Memory responses raised during a DENV infection modulate disease severity during a subsequent DENV challenge. Most primary (1°) DENV infections are subclinical or manifest as dengue fever and induce protective immunity against the same DENV serotype. In contrast, subsequent infection with a different DENV serotype may lead to potentially fatal dengue hemorrhagic fever/dengue shock syndrome, due to antibody-dependent enhancement (ADE) [27] and/or serotype cross-reactive T cells [28]. During ADE, antibodies from a previous DENV infection bind, but do not neutralize, the secondary DENV serotype, facilitate DENV infection of Fcγ-receptor expressing cells, and may thus increase disease severity [27], [29], [30].

By the time symptoms of dengue develop 4–8 days after the bite of a DENV-infected mosquito, the site of DENV transmission is no longer apparent. Therefore, biopsies of naturally DENV-infected human skin are not available, and animal models must serve to study dynamics of the immune response in the skin. DENV suppresses the interferon (IFN) response, replicates, and causes disease in humans but not wild-type (WT) mice [31]–[33]. Most DENV infection models use mice deficient in the IFN pathway, such as IFN-α/β and -γ receptor-deficient (AG129) mice that display virus tropism similar to humans and a vascular leak syndrome with key features of severe dengue disease [34], [35]. We recently improved this model by using the virulent DENV2 strain D220 in the less immunodeficient *Ifnar^–/–^* mice in the C57BL/6 background, which lack the IFN-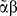 receptor but express functional IFN-γ receptor [36].

Here, we establish an intradermal (i.d.) DENV infection model in *Ifnar^–/–^* mice to study the early immune response during DENV infection of the dermis. Comparing cell populations in the steady-state dermis and performing adoptive transfers of WT or *Ifnar^–/–^* monocytes, we confirm the normal phenotype, frequency, and response of *Ifnar^–/–^* monocytes and DCs in the skin. Using Ly6C expression, we resolved the heterogeneity of dermal DCs. We find that dermal Ly6C^–^ CD11b^+^ cDCs and MΦs are the initial targets for DENV infection, but Ly6C^high^ monocytes are recruited to the dermis, differentiate to Ly6C^+^ CD11b^+^ moDCs, and become the major targets for DENV infection after 48 h. Our study unveils a novel viral strategy of exploiting monocyte recruitment to increase the targets for virus replication at the site of transmission in the skin.

## Results

### A novel model of intradermal DENV2 infection in *Ifnar* ^–/–^ mice

**Figure 1:**
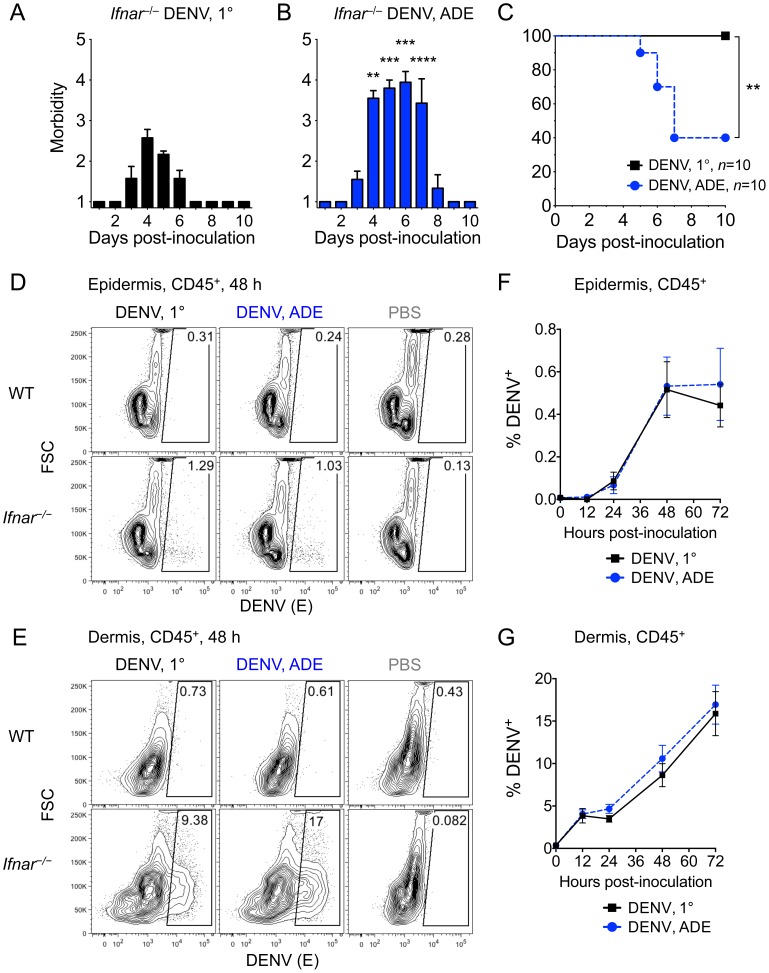
DENV2 infection of the skin in *Ifnar* ^–/–^ but not WT mice and lethal disease during ADE. (A and B) *Ifnar* ^–/–^ mice were injected i.d. with DENV2 under 1° (A) or ADE (B) infection conditions. Mean morbidity +SEM on a scale from 1 =  healthy to 5 =  moribund. The dotted line marks the time-point 72 h, when symptoms of disease appeared. (C) Survival of i.d. DENV2-infected *Ifnar* ^–/–^ mice (data pooled from 3 independent experiments, *n* = 10 per condition). Significant differences between 1° and ADE infection conditions are marked as *, *p*≤0.05; **, *p*≤0.01; ***, *p*≤0.001; and ****, *p*≤0.0001. (D and E) Flow cytometric analysis of FSC and intracellular DENV protein E in CD45^+^ hematopoietic cells from the epidermis (D) and dermis (E) of WT and *Ifnar* ^–/–^ mice 48 hours post-i.d. inoculation (hpi) with DENV2 or PBS (2 repeats, *n* = 6 per condition). (F and G) Time-courses showing mean ±SEM of CD45^+^ hematopoietic cells in the epidermis (F) or dermis (G) of *Ifnar* ^–/–^ mice staining positive for intracellular DENV proteins NS3 and E, 12–72 hpi with DENV2 under 1° (black square) or ADE (blue circle) infection conditions. Data are pooled from 2–5 repeats (*n* = 6–13 per time-point and condition). See also Figure S1.

To establish an intradermal DENV infection model, we evaluated DENV infection of the skin of WT and *Ifnar^–/–^* mice. We infected WT and *Ifnar* ^–/–^ mice i.d. with DENV2 under 1° or ADE conditions. As expected, DENV2 did not cause disease in WT mice after i.d. inoculation under either condition (Figure S1). In contrast, *Ifnar* ^–/–^ mice developed mild disease after i.d. inoculation with 10^6^ plaque-forming units (PFU) of DENV2 under 1° conditions and showed significantly more severe and prolonged disease under ADE conditions (Fig. 1, A and B). Whereas 1° infections were sublethal, 60% of *Ifnar* ^–/–^ mice succumbed to ADE infection 5-7 days post-i.d. inoculation (Fig. 1C) via a vascular leak-like syndrome, which displays features similar to severe dengue disease in humans [29].

Since little information exists on early DENV infection in the skin before onset of illness, we focused on the first 3 days of DENV infection (Fig. 1, A and B). No DENV E protein was detectable in CD45^+^ hematopoietic cells in the epidermis or dermis of WT mice (Fig. 1, D and E) 48 h post-i.d. inoculation (hpi) with 10^6^ PFU DENV2 under 1° or ADE infection conditions. In contrast, CD45^+^ cells in the epidermis and dermis of *Ifnar^–/–^* mice displayed DENV E staining (Fig. 1, D and E). We further stained skin samples from *Ifnar^–/–^* mice for intracellular DENV non-structural protein NS3, indicative of active viral replication [22]. No significant difference in DENV infection of total CD45^+^ cells in the skin of *Ifnar^–/–^* mice existed between 1° and ADE conditions (Fig. 1, F and G), implying that the differences in pathogenesis occurred later, after systemic viral spread. While the epidermis of *Ifnar^–/–^* mice contained <0.54% DENV-infected CD45^+^ cells (Fig. 1F), the dermis contained up to 17% DENV-infected CD45^+^ cells (Fig. 1G) and 3.3-fold more total CD45^+^ cells than the same area of epidermis. Hence, the dermis of *Ifnar^–/–^* mice contained ∼100-fold more DENV-infected CD45^+^ cells than the epidermis at 72 hpi.

### DENV2 infects few LCs in the epidermis

**Figure 2:**
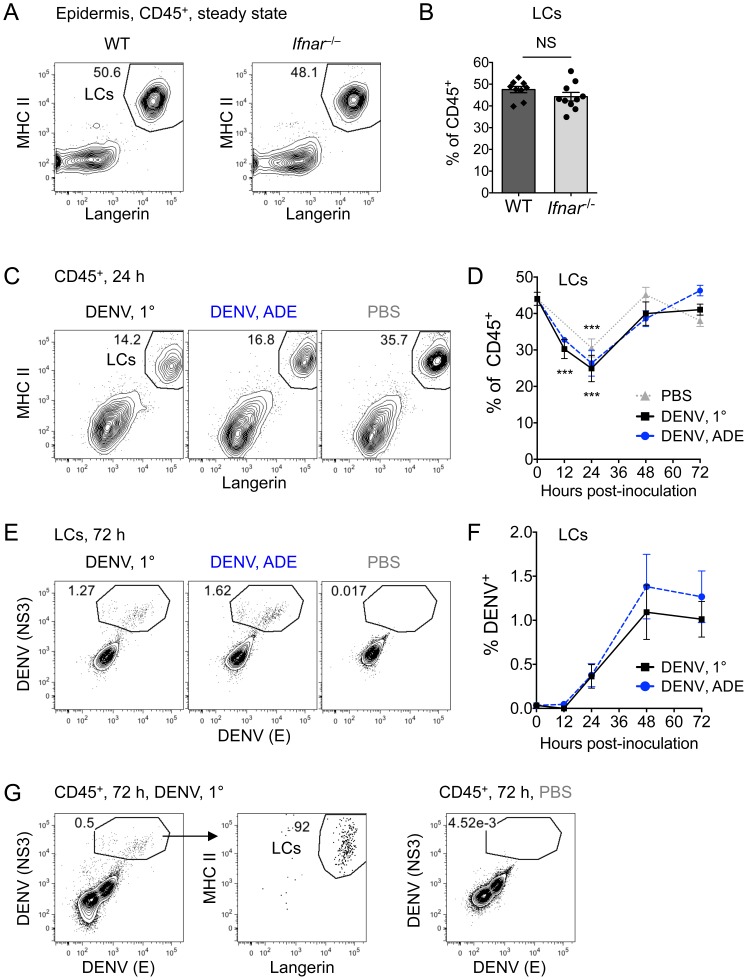
Frequency and DENV infection of LCs in the epidermis. (A) Gating of LCs from CD45^+^ cells in the epidermis of steady-state WT and *Ifnar* ^–/–^ mice. (B) Bar graph showing the mean ± standard error of the mean (SEM) and single data points of the % LCs among CD45^+^ cells in the epidermis. (C) Gating of LCs among CD45^+^ cells in the epidermis of *Ifnar* ^–/–^ mice 24 hpi with DENV2 under 1° or ADE conditions or after inoculation with PBS. (D) Time-course showing mean % LCs ±SEM in the epidermis after i.d. inoculation with DENV2 under 1° (black square) or ADE (blue circle) infection conditions or after inoculation with PBS (grey triangle). Time-point “0” is the frequency of LCs in the steady-state untouched epidermis. Significant differences in LC frequencies between steady state and i.d. inoculation are marked as ***, *p*≤0.001, and differences for 1° or ADE DENV2 infection are marked together as they were similar. (E) Intracellular staining of LCs for DENV proteins NS3 and E 72 hpi. (F) Time-course showing % DENV^+^ LCs in the epidermis during 1° (black square) and ADE (blue circle) DENV infection. (G) MHC II and Langerin expression of all CD45^+^ cells that were gated DENV NS3^+^ E^+^ 72 hpi under 1° conditions or with PBS. Representative plots or pooled data are from 2–5 independent experiments (*n* = 6–15 per time-point and condition). See also Figure S2.

Next, we set out to identify the cell types that DENV infects in the skin and first examined the epidermis more closely. We evaluated the frequency of LCs in the epidermis of WT and *Ifnar^–/–^* mice in steady state, gating LCs as CD45^+^ MHC II^+^ Langerin^+^ (Fig. 2A). The phenotype and frequency of LCs were similar in WT and *Ifnar* ^–/–^ mice, with LCs comprising ∼46% of CD45^+^ cells in both mouse strains (Fig. 2B). Twelve and 24 hpi with DENV, LC frequencies significantly decreased in the epidermis of *Ifnar^–/–^* mice, with a decline of 42% at 24 h (Fig. 2, C and D) compared to steady-state untouched ears. Primary and ADE infection conditions as well as inoculation with PBS reduced LCs frequencies similarly. This was likely caused by exit from the epidermis and migration to LNs, as DENV inoculation did not increase LC death in the epidermis. By 48 hpi, LC frequency recovered to steady-state levels (Fig. 2D). Staining for DENV proteins demonstrated infection of epidermal LCs (Figure 2E), which peaked 48–72 hpi and was similar under 1° and ADE conditions (Fig. 2F). LCs were the main DENV-infected hematopoietic cells in the epidermis, as over 90% of NS3^+^ E^+^ cells were LCs (Fig. 2G and Figure S2), and the remaining epidermal CD45^+^ cells that were mostly γδT cells were not DENV-infected. Our results in the *Ifnar^–/–^* model are in line with DENV2 inoculation of human skin explants that have found DENV infection of epidermal LCs [24], [25].

### Maintenance of hematopoietic cell populations in the steady-state dermis does not depend on IFN-α/β receptor signaling

**Figure 3:**
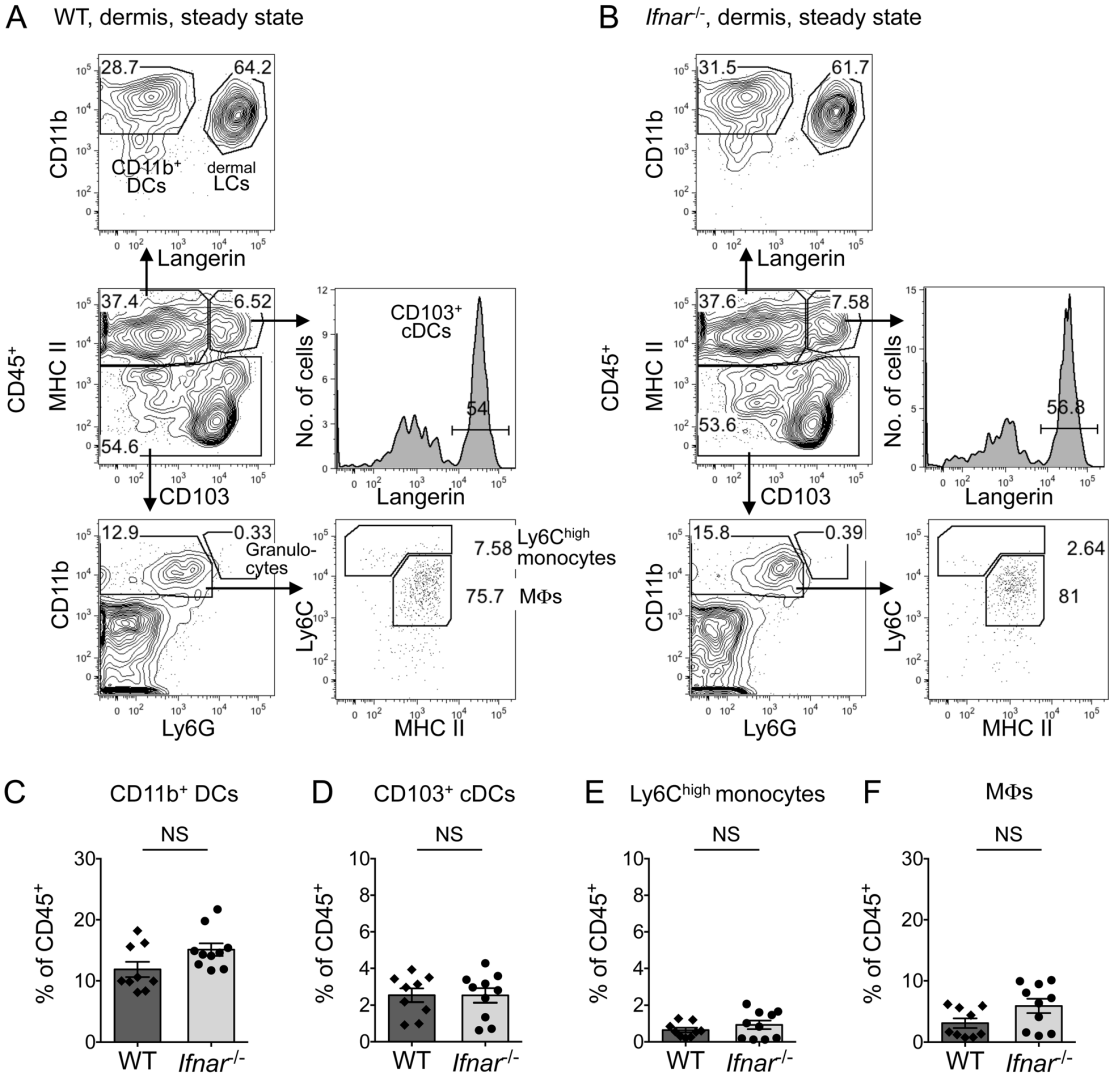
Normal cell populations in steady-state dermis of *Ifnar* ^–/–^ mice. (A and B) Consecutive gating of cell populations from CD45^+^ cells in the dermis of steady-state WT (A) and *Ifnar* ^–/–^ (B) mice. Depicted ciphers in the graphs are the % of cells in the gate relative to the parent gate. (C-F) Bar graphs showing mean ±SEM and single data points of the % CD11b^+^ DCs (C), CD103^+^ cDCs (D), and Ly6C^high^ monocytes (E), and MΦs (F), as gated in A and B, and calculated as frequency among all CD45^+^ cells in the steady-state dermis. Data are pooled from 3 independent experiments (*n* = 9–10 per mouse strain), and statistical comparisons between WT and *Ifnar* ^–/–^ mice are indicated (NS, non-significant). See also Figure S3.

We next focused on the dermis, evaluating DC, monocyte, and MΦ frequencies in the dermis of WT and *Ifnar^–/–^* mice in steady state. The dermis contained similar cell populations in steady-state WT (Fig. 3A) and *Ifnar* ^–/–^ mice (Fig. 3B). We separated dermal CD45^+^ cells by MHC II expression into MHC II^high^ DCs and MHC II^low/–^ non-DCs. The main dermal population was CD11b^+^ DCs (on average ∼14% of all CD45^+^ cells, Fig. 3C), gated as MHC II^high^ CD103^–^ CD11b^+^ Langerin^–^. Additionally, the dermis contained MHC II^+^ CD103^–^ CD11b^+^ Langerin^+^ cells, which were likely dermal LCs migrating from the epidermis through the dermis to LNs [37]. Both WT and *Ifnar* ^–/–^ mice contained dermal CD103^+^ cDCs (∼2.5% of CD45^+^ cells; Fig. 3D), gated as MHC II^high^ CD103^+^ Langerin^+^. The steady-state dermis contained only 0.87% Ly6C^high^ monocytes of all CD45^+^ cells (Fig. 3E), gated as MHC II^–^ CD11b^+^ Ly6G^–^ Ly6C^high^, and did not contain MHC II^–^ CD11b^+^ Ly6G^+^ granulocytes. Dermal MΦs were MHC II^low/–^ CD11b^+^ Ly6G^–^ Ly6C^low/–^, expressed F4/80 and a high FSC/SSC profile (Figure S3), and constituted ∼4.6% of CD45^+^ cells (Fig. 3F). The hematopoietic cells in the skin that did not stain positive for the indicated markers mostly consisted of T cells expressing the γδT-cell receptor, other T cells, or mast cells. No statistically significant difference existed between WT and *Ifnar* ^–/–^ mice in any cell population examined in the steady-state skin (Fig. 3C–F). These results indicate that IFNAR signaling is not required to maintain hematopoietic cells in the steady-state skin and substantiate *Ifnar* ^–/–^ mice as a model to study immune cells in the skin. We continued to examine DENV infection in the dermis of *Ifnar^–/–^* mice because WT mice did not develop disease or support DENV2 replication, but *Ifnar^–/–^* mice carried equivalent immune cell populations in steady-state skin, supported DENV infection, and developed key features of human dengue disease.

### A marked increase in monocytes & moDCs in the DENV-infected dermis coincides with a decrease in cDCs & MΦs

**Figure 4:**
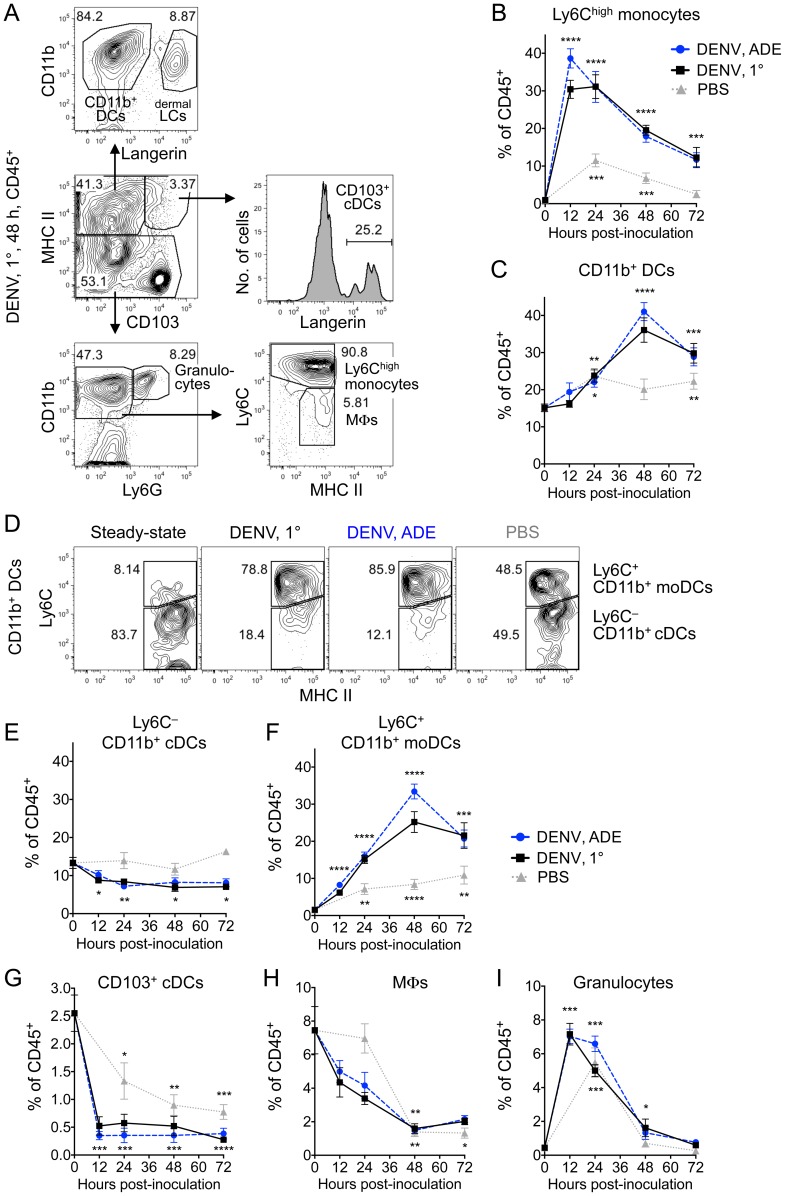
Dynamics of cell populations in the DENV-infected dermis. (A) Gating of cell populations in the dermis in *Ifnar* ^–/–^ mice 48 hpi with DENV2 under 1° infection conditions. (B and C) Time-courses showing mean ±SEM of % Ly6C^high^ monocytes (B) and CD11b^+^ DCs (C) among dermal CD45^+^ cells after i.d. inoculation with DENV2 under 1° (black square) or ADE (blue circle) infection conditions or after inoculation with PBS (grey triangle). Time-point “0” is the frequency of each cell type in steady-state untouched dermis (data from Fig. 1). (D) Ly6C expression separating CD11b^+^ DCs into Ly6C^+^ CD11b^+^ moDCs and Ly6C^–^ CD11b^+^ cDCs in the steady-state dermis, or 48 hpi with DENV2 or PBS. (E–I) Time-courses showing mean ±SEM of % Ly6C^–^ CD11b^+^ cDCs (E), Ly6C^+^ CD11b^+^ moDCs (F), CD103^+^ cDCs (G), MΦs (H), and granulocytes (I) among dermal CD45^+^ cells. Significant differences between cell frequencies in steady state and after i.d. inoculation are marked as * for *p*≤0.05; **, *p*≤0.01; ***, *p*≤0.001; and ****, *p*≤0.0001. Asterisks designate *p*-values comparing steady state to 1° or ADE DENV2 infection together as they were similar. Representative plots or pooled data are from 2–4 independent experiments (*n* = 6–10 per time-point and condition). See also Figure S4.

No information existed on the cells that first encounter DENV in the dermis and the dynamics of the DENV-induced immune response. We monitored dermal cell populations after i.d. inoculation with DENV2 or PBS in *Ifnar^–/–^* mice (Fig. 4A and Figure S4). Notably, dermal Ly6C^high^ monocytes increased significantly during DENV infection compared to steady-state untouched ears and peaked after only 12 h (Fig. 4B). The increase of Ly6C^high^ monocytes was significantly higher during ADE compared to 1° infection conditions (*p*<0.05 at 12 hpi) with a 39- and 30-fold peak increase, respectively. PBS-injected controls showed some increase of Ly6C^high^ monocytes compared to steady state, but significantly less than during DENV infection (24 h, *p*<0.01; 48 h, *p*<0.0001; and 72 h, *p*<0.01).

Furthermore, CD11b^+^ DCs increased significantly, ∼2.5-fold in the dermis 48 hpi with DENV (Fig. 4C). As Ly6C^high^ monocytes enter inflamed tissues and give rise to moDCs [15], [38], [39], we hypothesized that moDCs contributed to the increase in CD11b^+^ DCs in the DENV-infected dermis. Dermal CD11b^+^ DCs were heterogeneous and, in line with recent studies [16], [17], could be separated based on Ly6C expression (Fig. 4D and Table 1). The steady-state dermis contained mostly Ly6C^–^ CD11b^+^ cDCs, which decreased 24 hpi with DENV2 (Fig. 4E). In contrast, Ly6C^+^ CD11b^+^ moDCs significantly increased and peaked at 48 h (Fig. 4F). The increase of Ly6C^+^ CD11b^+^ moDCs was significantly higher during ADE compared to 1° infection conditions (*p*<0.05 at 48 hpi) with a 21- and 16-fold peak increase, respectively. PBS-injected controls showed some increase of Ly6C^+^ CD11b^+^ moDCs (Fig. 4F), but significantly less than during DENV infection (24 h, *p*<0.01; 48 h, *p*<0.0001; and 72 h, *p*<0.05). Ly6C expression thus resolved the heterogeneity of CD11b^+^ DCs into Ly6C^–^ CD11b^+^ cDCs resident in steady-state dermis and Ly6C^+^ CD11b^+^ moDCs that increased during DENV infection.

**Table 1:** Gating of hematopoietic cell populations in the dermis.

Name of subset	Gated phenotype		
Ly6C^high^ monocytes	CD45^+^ MHC II^low/–^		CD11b^+^ Ly6G^–^ Ly6C**^high^**
Ly6C^+^ CD11b^+^ moDCs	CD45^+^ MHC II**^high^**	CD103^–^ Langerin^–^	CD11b^+^ Ly6G^–^ Ly6C**^+^**
Ly6C^–^ CD11b^+^ cDCs	CD45^+^ MHC II**^high^**	CD103^–^ Langerin^–^	CD11b^+^ Ly6G^–^ Ly6C^–^
CD103^+^ cDCs	CD45^+^ MHC II**^high^**	CD103**^+^** Langerin**^+^**	
Dermal LCs	CD45^+^ MHC II**^high^**	CD103^–^ Langerin**^+^**	CD11b^+^
MΦs	CD45^+^ MHC II^low/–^		CD11b^+^ Ly6G^–^ Ly6C^low^
Granulocytes	CD45^+^ MHC II^low/–^		CD11b^+^ Ly6G**^+^**

In addition, CD103^+^ cDCs (Fig. 4G) and MΦs (Fig. 4 H) significantly decreased in the dermis by 12 and 48 hpi with DENV2, respectively, and remained low through 72 h. PBS-injected controls also showed reduction of CD103^+^ cDCs and MΦs, but less pronounced than after inoculation with DENV2 (at 24 hpi, *p*<0.05 for CD103^+^ cDCs and *p*<0.01 for MΦs). In contrast, granulocytes significantly increased 16-fold after i.d. inoculation with DENV2, showing a temporary peak after 12–24 h, but then decreased (Fig. 4I). No increased cell death was observed in the dermis after inoculation with DENV, suggesting that the decreased number of cDCs was due to exit of cDCs from the dermis and migration to LNs. Overall, the immune response in the dermis was slightly more pronounced during ADE compared to 1° DENV infection. The increase in dermal Ly6C^high^ monocytes and moDCs, as well as decrease in cDCs and MΦs, likely impacted the number of targets for DENV replication.

### Classical DCs and MΦs are the initial targets of DENV replication in the dermis, followed by monocytes and moDCs that become the main targets in a second wave of infection

**Figure 5:**
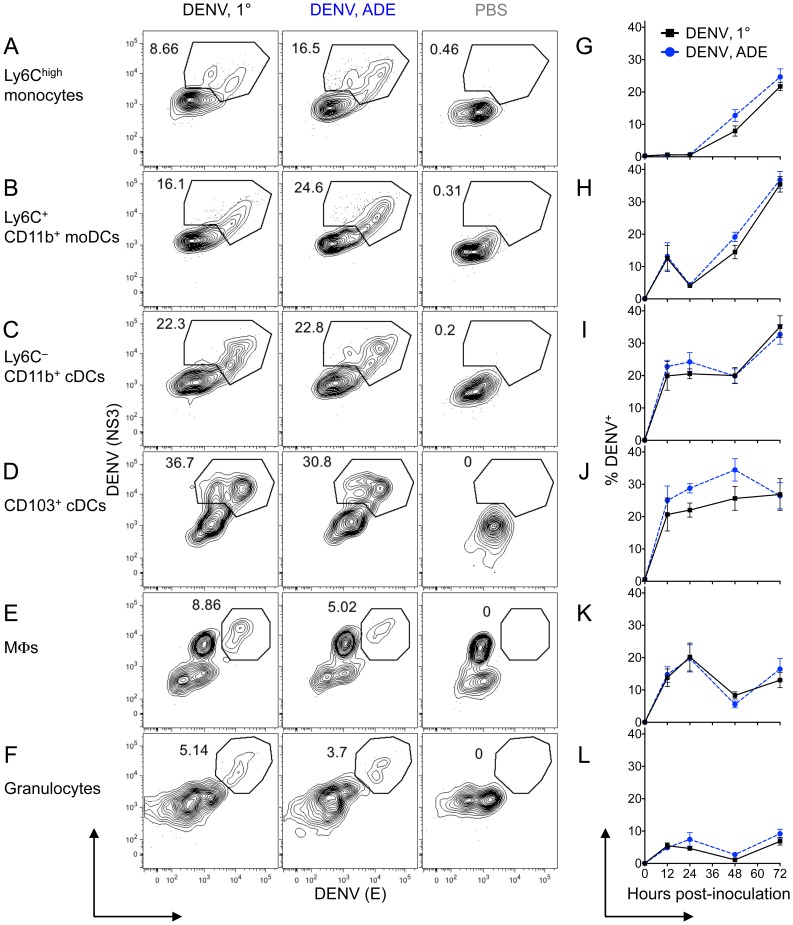
DENV infection of monocytes, DCs, and MΦs in the dermis. (A–F) *Ifnar* ^–/–^ mice were inoculated i.d. with DENV2 during 1° or ADE infection or with PBS. Intracellular staining for DENV proteins NS3 and E is shown after 48 h. (G–L) Time-course of mean ±SEM of % NS3^+^ E^+^ DENV-infected cells for each population in the dermis under 1° (black square) or ADE (blue circle) infection conditions. Ly6C^high^ monocytes (A and G), Ly6C^+^ CD11b^+^ moDCs (B and H), Ly6C^–^ CD11b^+^ cDCs (C and I), CD103^+^ cDCs (D and J), MΦs (E and K), and granulocytes (F and L) were gated as in Fig. 4. Representative plots or pooled data are from 2–4 independent experiments (*n* = 6–12 per time-point and condition).

Previous studies examined DENV infection only in the epidermis. Here, we analyzed DENV infection via intracellular staining of DENV proteins E and NS3 in the dermis (Fig. 5A–F). Dermal Ly6C^high^ monocytes (Fig. 5G) and Ly6C^+^ CD11b^+^ moDCs (Fig. 5H) showed no or low levels of DENV infection 12–24 hpi, but became DENV-infected by 48 hpi and then steeply increased DENV infection, up to 37% for moDCs at 72 h. In contrast, more than 20% of Ly6C^–^ CD11b^+^ cDCs, CD103^+^ cDCs, and MΦs became DENV-infected within the first 12–24 h (Fig. 5I-K). While DENV infection of Ly6C^–^ CD11b^+^ cDCs and CD103^+^ cDCs remained high throughout (20–34%), DENV infection of MΦs declined after 24 h (Fig. 5K). Less than 10% of granulocytes showed DENV infection at all times (Fig. 5L). A trend of higher DENV infection during ADE compared to 1° conditions appeared at some time-points and populations, but no significant difference existed (Fig. 5G–L). For the first time, we show that Ly6C^–^ CD11b^+^ cDCs, CD103^+^ cDCs, and MΦs, which are present in the steady-state dermis, are the initial targets for DENV replication. During a second wave of infection, dermal Ly6C^high^ monocytes and Ly6C^+^ CD11b^+^ moDCs become highly DENV-infected.

**Figure 6:**
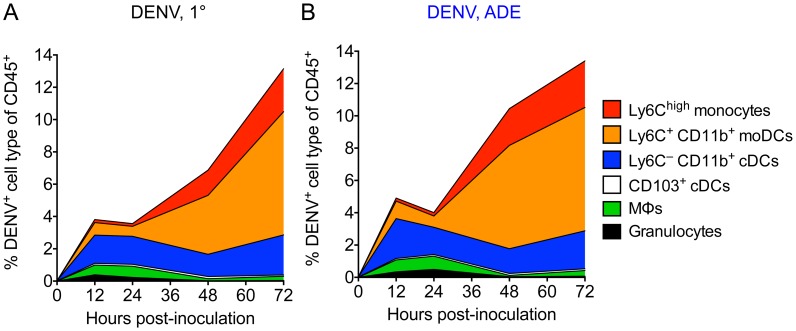
The main targets for DENV infection in the dermis over time. (A and B) Stacked area charts show mean distribution of all DENV^+^ CD45^+^ cells among cell populations in the dermis 12, 24, 48, and 72 hpi with DENV2 under 1° (A) or ADE (B) infection conditions. Individual populations from top to bottom: Ly6C^high^ monocytes, Ly6C^+^ CD11b^+^ moDCs, Ly6C^–^ CD11b^+^ cDCs, CD103^+^ cDCs, MΦs, and granulocytes. The depicted area for each population represents the % DENV-infected cells for this cell type of CD45^+^ cells. Data are pooled from 2-4 independent experiments (*n* = 6–12 per time-point and condition).

The abundance of a certain cell type together with its susceptibility to virus infection determines its contribution to virus replication. We multiplied the frequency of a dermal cell type (Fig. 4) by its percent DENV infection (Fig. 5) to determine its contribution to overall infection (Fig. 6). DENV-infected Ly6C^–^ CD11b^+^ cDCs contributed to virus replication at all times, representing ∼1.5–2.5% of total CD45^+^ cells 12–72 hpi (Fig. 6). In contrast, DENV-infected MΦs contributed 0.8% of total CD45^+^ cells during the first 24 h, but then waned. Although infected, CD103^+^ cDCs did not contribute considerably to DENV replication at any time-point examined (≤0.1%) due to their low frequency after inoculation with DENV. Classical DCs and MΦs that reside in the steady-state dermis thus were the main DENV-infected cells in the initial phase, up to 24 hpi.

In a second phase, 48–72 hpi, monocytes and moDCs became the main targets of DENV replication in the dermis (Fig. 6). While Ly6C^high^ monocytes were present in high numbers as early as 12 hpi (Fig. 4B), they became DENV-infected only after 48 h and then contributed ∼2.3% of CD45^+^ cells (Fig. 6). Ly6C^+^ CD11b^+^ moDCs showed substantial DENV infection starting at 48 hpi, when they peaked in frequency (Fig. 4F), and DENV-infected moDCs constituted 7.6% of total CD45^+^ cells at 72 hpi (Fig. 6). The mean % CD45^+^ cells consisting of DENV-infected Ly6C^+^ CD11b^+^ moDCs was significantly larger during ADE (mean 6.4%±0.75 SEM) compared to 1° infection conditions (3.8%±0.61) at 48 hpi (*p*<0.05, unpaired *t*-test). Together, DENV-infected monocytes and moDCs made up 79% of all infected cells by 72 hpi and were thus the major targets for virus replication in a second wave of infection.

### Adoptively-transferred CCR2^+^ Ly6C^high^ monocytes are recruited to the inflamed dermis, differentiate to Ly6C^+^ CD11b^+^ moDCs, and become DENV-infected

**Figure 7:**
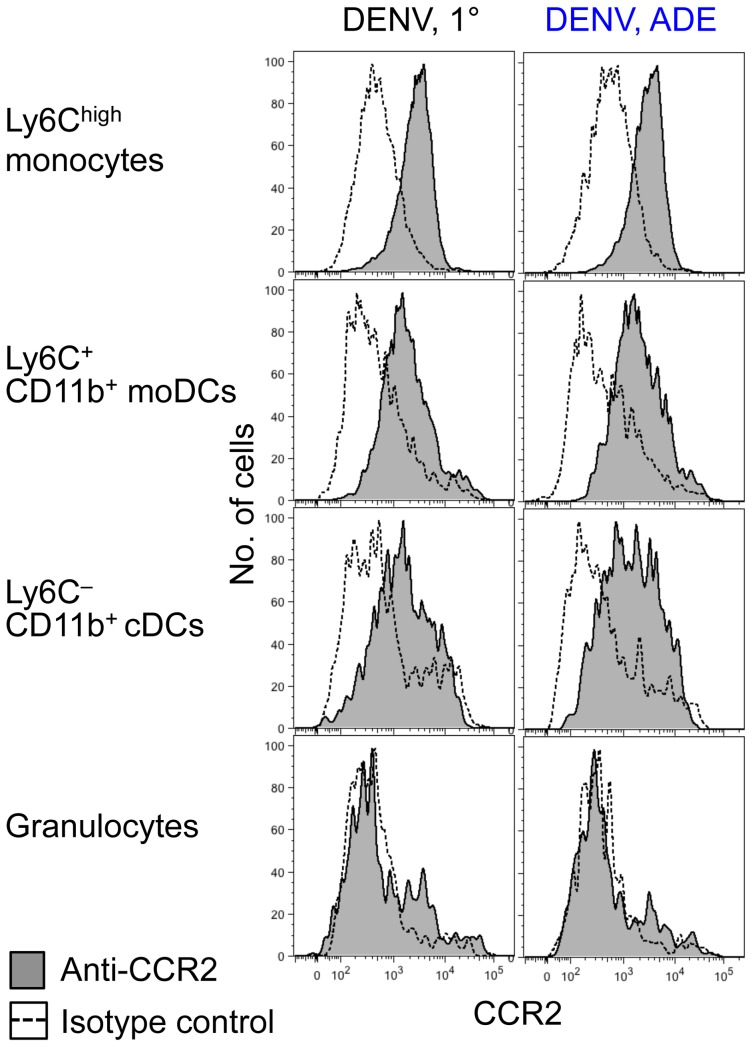
CCR2 expression on Ly6C^high^ monocytes, moDCs, and cDCs. *Ifnar* ^–/–^ mice were inoculated i.d. with DENV2 during 1° or ADE infection. CCR2 expression compared to isotype-matched control stains of Ly6C^high^ monocytes, Ly6C^+^ CD11b^+^ moDCs, Ly6C^–^ CD11b^+^ cDCs, and granulocytes 24 hpi with DENV under 1° or ADE conditions. Representative data from two independent experiments (*n* = 6 per condition).

Next, we aimed to determine the mechanism of how monocytes and moDCs increase in the DENV-infected dermis. To test the hypothesis that Ly6C^high^ monocytes enter the DENV-infected dermis, differentiate to Ly6C^+^ CD11b^+^ moDCs, and sustain DENV infection, we determined CCR2 expression, which has been shown to be essential for repopulation of dermal DCs in the inflamed skin [40], and performed adoptive transfer experiments. Ly6C^high^ monocytes and Ly6C^+^ CD11b^+^ moDCs in the DENV-infected dermis expressed chemokine receptor CCR2 on their surface 24 hpi with DENV under 1° or ADE conditions (Fig. 7), similar to Ly6C^–^ CD11b^+^ cDCs, which is in line with previous studies [17]. In contrast, granulocytes that were also recruited to the dermis did not express CCR2. These results suggest that Ly6C^high^ monocytes may be recruited to the DENV-infected dermis via signaling through CCR2, while other migratory markers mediate the recruitment of granulocytes.

**Figure 8:**
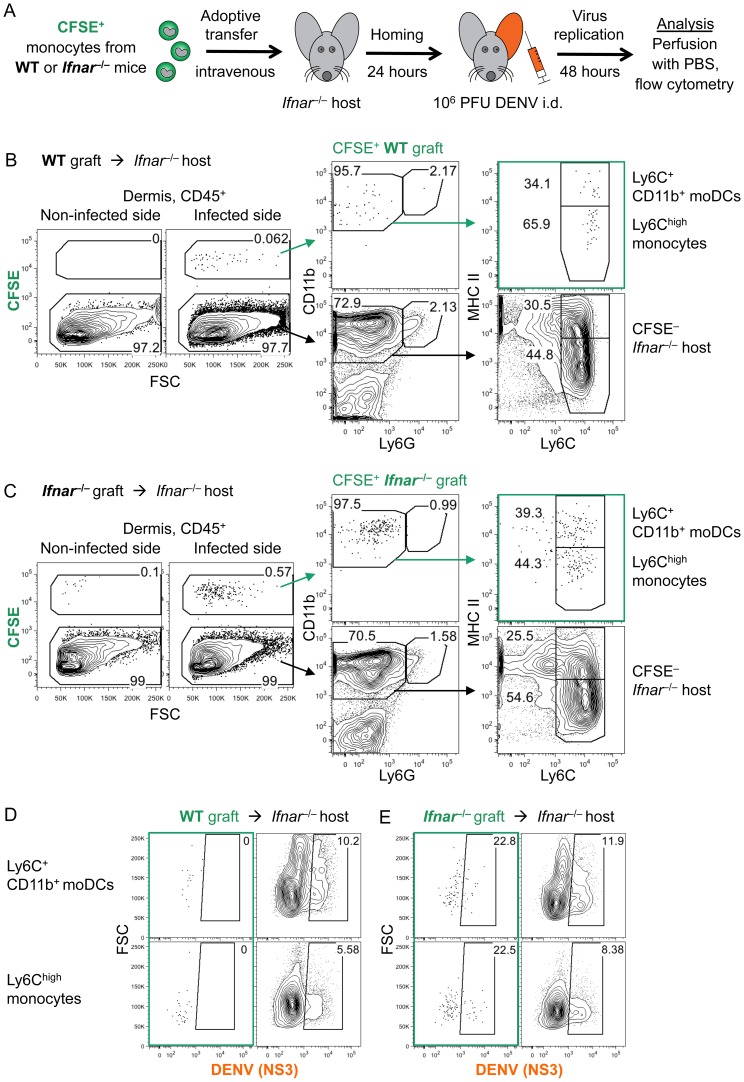
Ly6C^high^ monocytes enter the DENV-infected dermis and differentiate to moDCs. (A) Ly6C^high^ monocytes were isolated from the bone marrow of steady-state WT and *Ifnar* ^–/–^ mice, labeled with CFSE, and transferred intravenously into 4 week-old *Ifnar* ^–/–^ recipients. After 24 h, one ear was infected with DENV2 under 1° conditions, while the other ear remained untouched. Ear skin was analyzed 48 hpi with DENV2. (B and C) Recipients obtained 6×10^6^ monocytes from WT (B) or 9×10^6^ monocytes from *Ifnar* ^–/–^ donors (C). Gating of CFSE^+^ graft and CFSE^–^ host cells among CD45^+^ cells in the dermis of the infected or non-infected side. CD11b^+^ Ly6G^–^ Ly6C^+^ and MHC II^–^ monocytes or MHC II^+^ moDCs were gated as indicated. (D and E) DENV NS3 intracellular staining of Ly6C^+^ CD11b^+^ moDCs or Ly6C^high^ monocytes in CFSE^+^ WT graft (D), CFSE^+^ *Ifnar* ^–/–^ graft (E), or CFSE^–^ *Ifnar* ^–/–^ host cells (D and E). Representative plots of 2 independent experiments (*n* = 10 total recipients and *n* = 4 non-transplanted controls). See also Figure S5.

Next, we isolated monocytes from the bone marrow of steady-state WT or *Ifnar* ^–/–^ mice via negative magnetic-bead selection, yielding purities of 91–95% and a Ly6C^high^ CCR2^high^ phenotype (Figure S5). Monocytes were labeled with CFSE, transferred intravenously into *Ifnar* ^–/–^ hosts, and allowed to home for 24 h. Then, recipients were inoculated i.d. with DENV2 into one ear under 1° infection conditions (Fig. 8A). Before extracting skin samples for analysis, euthanized recipients were perfused with PBS to exclude cells circulating in the blood from the samples. Mice that were infected with DENV2 but had not received monocyte transfers served as gating controls for CFSE^+^ graft-derived cells.

Adoptively transferred CFSE^+^ Ly6C^high^ monocytes from WT (Fig. 8B) or *Ifnar* ^–/–^ (Fig. 8C) origin engrafted robustly into the dermis of the DENV-infected ear of *Ifnar* ^–/–^ hosts 48 hpi. In contrast, the dermis of control steady-state recipients (Figure S5) or the non-infected side of DENV-infected recipients (Fig. 8, B and C) showed minimal engraftment. No monocyte engraftment was detected in the epidermis. These results provide direct evidence that monocytes were specifically recruited and entered the DENV-infected dermis.

Further, 34% of engrafted monocyte-derived WT cells (Fig. 8B) and 39% of *Ifnar* ^–/–^ cells (Fig. 8C) in the DENV-infected dermis expressed MHC II and Ly6C and had thus differentiated to Ly6C^+^ CD11b^+^ moDCs. The remaining graft was still Ly6C^high^ monocytes. Therefore, the recruitment and differentiation of WT and *Ifnar* ^–/–^ monocytes during early DENV infection was equivalent and thus independent of IFNAR signaling.

Although, as expected, WT cells were not infected (Fig. 8D), approximately 22% of *de novo*-recruited monocytes and moDCs that derived from the *Ifnar* ^–/–^ graft were DENV-infected 48 hpi (Fig. 8E). We show for the first time that the increase of Ly6C^high^ monocytes in the DENV-infected dermis is due to specific recruitment from the blood and entry into the DENV-infected dermis, which was likely mediated by CCR2. We further demonstrate the differentiation of Ly6C^high^ monocytes to Ly6C^+^ CD11b^+^ moDCs and DENV infection of *de novo-*recruited cells, leading to the increase in targets for DENV replication in the dermis.

### Non-infected bystander monocytes, moDCs and cDCs express higher levels of activation markers CD80 and CD86 than DENV-infected cells in the dermis

**Figure 9:**
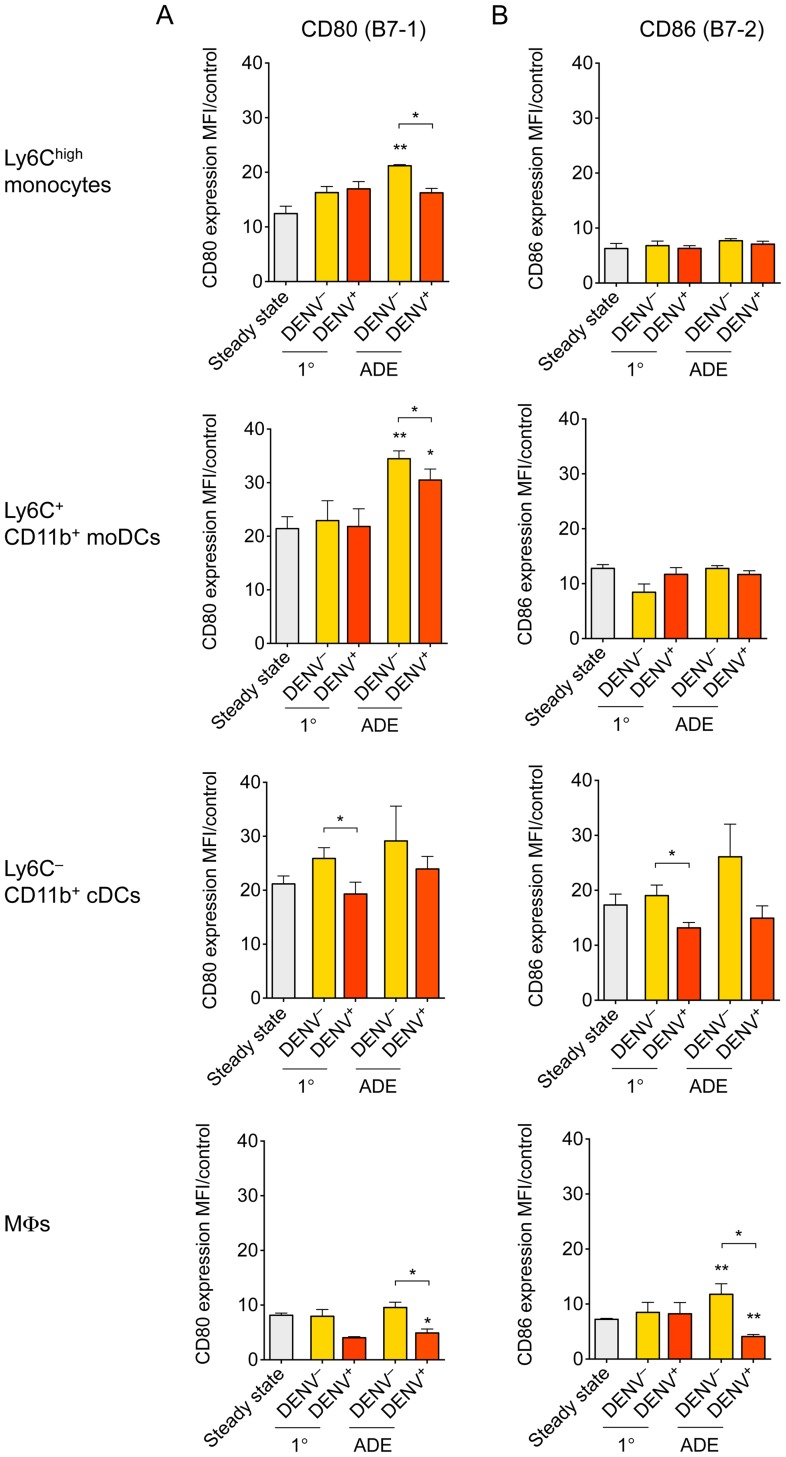
Expression of activation markers on Ly6C^high^ monocytes, moDCs, cDCs and MΦs. *Ifnar* ^–/–^ mice were inoculated i.d. with DENV2 during 1° or ADE infection. (A and B) Bar graphs show mean ±SEM of CD80 (B7-1) (A) or CD86 (B7-2) (B) surface protein expression determined via flow cytometric analysis and calculated as median fluorescence intensities (MFI) of specific stains divided by the MFI of isotype-matched control stains. CD80 and CD86 expression was examined on Ly6C^high^ monocytes, Ly6C^+^ CD11b^+^ moDCs, Ly6C^–^ CD11b^+^ cDCs, and MΦs during steady state, or 48 hpi with DENV under 1° or ADE conditions. In DENV-inoculated samples, each cell population was gated further on the presence of intracellular DENV proteins NS3 and E for DENV-infected cells (DENV^+^) or the absence of DENV proteins for non-infected bystander cells (DENV^–^). Significant differences in activation marker expression after i.d. inoculation with DENV compared to steady state are marked as * for *p*≤0.05 and ** for *p*≤0.01 above individual bars. Significant differences between DENV^–^ and DENV^+^ within the same sample are marked between bars with brackets and were calculated using the paired *t*-test. Pooled data from two independent experiments (*n* = 6 per condition).

Lastly, we examined the activation state of immune cells in the DENV-infected dermis, comparing 1° versus antibody-enhanced DENV infection to steady-state control mice. Relative to isotype-matched control stains, Ly6C^+^ CD11b^+^ moDCs and Ly6C^–^ CD11b^+^ cDCs expressed substantial amounts of the activation marker CD80 (B7-1) on the surface already in steady state, whereas Ly6C^high^ monocytes and MΦs expressed lower levels of CD80 (Fig. 9A). Forty-eight hours after DENV infection, non-infected bystander monocytes, Ly6C^+^ CD11b^+^ moDCs, and Ly6C^–^ CD11b^+^ cDCs upregulated CD80 in the dermis, whereas upregulation of CD80 was diminished in DENV-infected cells (Fig. 9A). The upregulation of CD80 in the dermis was more pronounced during ADE than during primary DENV infection. Steady-state and non-infected bystander MΦs expressed relatively low levels of CD80, and DENV-infected MΦs significantly down-regulated CD80 expression compared to steady-state or non-infected bystander cells. We also measured CD86 (B7-2) surface expression and observed similar trends as for CD80 expression on Ly6C^–^ CD11b^+^ cDCs and MΦs, i.e., that DENV-infected cells expressed significantly lower levels of CD86 than non-infected bystander cells (Fig. 9B). Our results show that DENV-infected monocytes, DCs, and MΦs express lower levels of activation markers than non-infected bystander DCs in the dermis. This confirms earlier studies that showed that DENV blocks the activation of DENV-infected human moDCs compared to non-infected bystander cells *in vitro* [41], [42].

## Discussion

**Figure 10:**
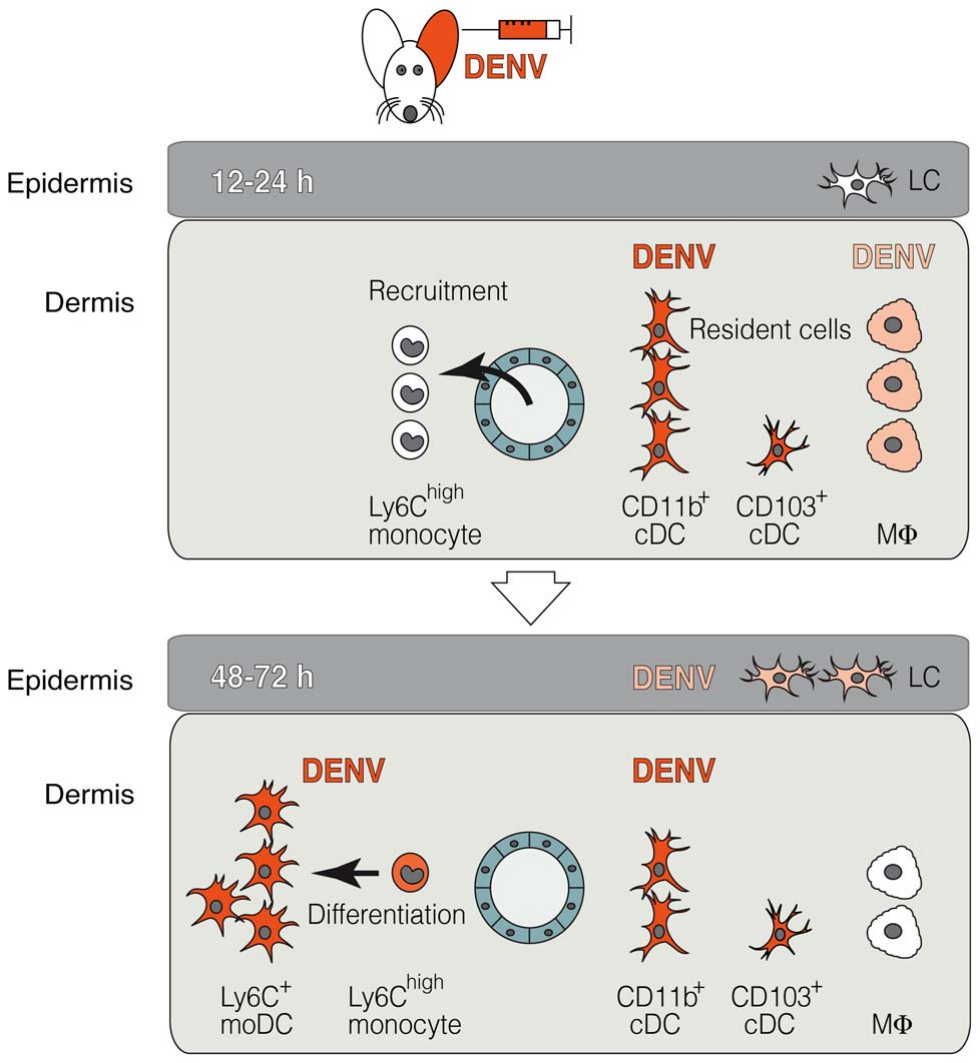
The two waves of DENV infection in the skin. Using a novel i.d. DENV infection model in the ear of *Ifnar* ^–/–^ mice, DENV was shown to initially infect resident cells of the dermis, most importantly Ly6C^–^ CD11b^+^ cDCs at 12–24 hpi. DENV also infects dermal CD103^+^ cDCs and to a lower extent MΦs, which decrease in frequency compared to steady state (top panel). At the same time, Ly6C^high^ monocytes are recruited from the blood to the DENV-infected dermis, where they differentiate to Ly6C^+^ CD11b^+^ moDCs, which become the major targets for DENV infection in a second wave of replication between 48–72 hpi (bottom panel). DENV infects approximately 100-fold more hematopoietic cells in the dermis than LCs in the epidermis.

While several subsets of DCs in the steady-state skin have been identified, many questions about pathogen invasion via the skin remain unanswered. Here we studied the immune response during DENV infection in the dermis. We identify resident cDCs and MΦs as initial targets for DENV replication and show that the recruitment of monocytes to the dermis and differentiation to moDCs greatly increased the number of targets for DENV replication in a second wave, as summarized in Fig. 10.

Infected *Aedes* mosquitoes transmit DENV when probing for blood vessels in the skin, yet few studies have examined DENV infection in the skin. DENV was shown to infect DCs in human skin explants, but epidermal LCs were not distinguished from dermal DCs [24]. Inoculating the surface of human skin explants with DENV later established infection of epidermal LCs [25], which was confirmed in a mouse model [26]. No information, however, existed about DENV infection in the dermis. Mosquitoes likely deposit DENV into the dermis when probing for blood, as the dermis, but not the epidermis, contains vasculature. We performed i.d. inoculation of DENV that was produced in C6/36 mosquito cells into the ears of mice to mimic the natural route of transmission. While confirming low levels of DENV infection of epidermal LCs, we establish that DENV infects ∼100-fold more CD45^+^ cells in the dermis than the epidermis, stimulating future studies to focus on the dermis as a major site for initial DENV replication.

In addition, factors in the saliva of the mosquito vector may impact DENV infection and the host response in the skin. For example, saliva from *Ae. aegypti* mosquitoes decreased DENV infection of moDCs *in vitro* [43]. In contrast, two mouse models showed increased or prolonged DENV serum viremia when DENV was inoculated in the presence of mosquito saliva [44], [45]. Clearly, further studies are required to determine the role of mosquito-derived factors in early DENV infection in the skin.

Investigating the immune cell network in the dermis is important to identify the cells that first encounter DENV. We extended recent advances in surface markers that characterize immune cell subsets in the steady-state dermis [11] and further dissect the DC network in the dermis to identify the cells that first encounter DENV. Combining MHC II and CD11b staining with Ly6G and Ly6C was important for dissecting Ly6G^+^ Ly6C^+^ granulocytes from Ly6G^–^ Ly6C^high^ monocytes and DC populations––and staining for Gr-1, which detects both Ly6G and Ly6C, would not have been sufficient. Similarly, Langerin staining was necessary to separate dermal Langerin^+^ CD103^+^ cDCs from Langerin^+^ CD11b^+^ LCs and Langerin^–^ CD11b^+^ DCs. We further dissected dermal CD11b^+^ DCs according to Ly6C expression into Ly6C^–^ CD11b^+^ cDCs and Ly6C^+^ CD11b^+^ moDCs during DENV infection, which is in line with a recent study showing that CD64^–^ Ly6C^–^ CD11b^+^ cDCs are different from CD64^+^ Ly6C^+^ CD11b^+^ moDCs in steady state, the latter of which clustered with Ly6C^high^ monocytes in gene expression analysis [17]. The combination of 7 cellular markers and 2 DENV intracellular proteins established Ly6C as valuable marker to dissect dermal CD11b^+^ DCs and allowed us to identify Ly6C^–^ CD11b^+^ cDCs, CD103^+^ cDCs, and MΦs that reside in the steady-state dermis as the initial targets for DENV replication in the skin. Knowing the initial targets of DENV infection may help develop therapeutic strategies to block DENV from establishing infection.

In steady state, Ly6C^high^ monocytes give rise to some CD11b^+^ DCs in non-lymphoid tissues [11], [46], but not in lymphoid tissues [47]. After depletion of CD11b-expressing cells, first Ly6C^high^ monocytes reappeared in the dermis, then after 7 days came Ly6C^high^ CD11b^+^ moDCs, and after 20 days Ly6C^low^ CD11b^+^ moDCs [17]. This suggested that few monocytes continuously enter the steady-state dermis and differentiate to moDCs. During DENV infection, we found here a large increase in dermal Ly6C^high^ monocytes already at 12 hpi. Adoptive transfers provided direct evidence that Ly6C^high^ monocytes are recruited to the DENV-infected dermis and rapidly differentiate to Ly6C^+^ CD11b^+^ moDCs. More than 90% of engrafted monocytes and moDCs in the dermis retained Ly6C expression 72 h after adoptive transfer of Ly6C^high^ monocytes. While activation may lead to the upregulation of Ly6C in some skin-resident cells, the dynamics of monocyte recruitment and differentiation to Ly6C^+^ CD11b^+^ moDCs suggest that most Ly6C-expressing cells in the DENV-infected dermis were monocyte-derived. These results are consistent with a parasite infection where Ly6C^high^ monocytes were recruited to *L. major*-infected dermis, differentiated to Ly6C^+^ CD11b^+^ moDCs, and became targets for infection [16]. Further, monocytes were found to replenish skin DCs during herpes simplex virus-1 infection [48], but monocytes and DCs are not the major targets for HSV infection. During human immunodeficiency virus-1 (HIV) infection, DCs produce chemokines that attract CD4^+^ T cells, which then serve as targets for HIV replication [49], [50]. Although DCs bind and shuttle HIV to CD4^+^ T cells for infection [51], HIV does not efficiently replicate in monocytes and DCs [49], [50]. In our study, *de novo*-recruited monocytes and moDCs became DENV-infected. To the best of our knowledge, our report is the first demonstration of a viral infection that uses the dermis as a primary port of entry and targets *de novo*-recruited monocytes and moDCs for local replication.

CCR2 and its ligand CCL2 are required for the mobilization of monocytes from the bone marrow [52] and recruitment to the skin during murine cytomegalovirus infection [53] and after exposure to ultraviolet light [40] or chemical irritants [17]. In dengue patients, CCL2 was increased in the plasma and positively correlated with disease severity [54]. Here, CCR2 was present on the surface of Ly6C^high^ monocytes and moDCs that were recruited to the DENV-infected dermis and likely mediated the recruitment. Nevertheless, further studies need to determine whether chemokine ligands for CCR2 are upregulated in the DENV-infected dermis, and whether CCR2 is required for the recruitment of Ly6C^high^ monocytes. Alternatively, CCR6 may play a role, although this is controversial, as CCR6 was found to be important for the recruitment of monocytes into the dermis after application of chemical adjuvants [55], but not for the repopulation of dermal DCs in the inflamed skin after exposure to ultraviolet light [40].

Exit from the skin, recruitment of precursors, and cell death determine the abundance of cell types that can serve as targets for virus replication. We did not observe increased cell death during DENV infection, and thus the decrease in dermal cDCs was likely due to exit from the dermis and migration to skin-draining LNs. DC migration from the skin to LNs is well established after exposure to ultraviolet light [40], chemical irritants [5], and during infection with herpes simples virus-1 [48], [56]. Pre-cDCs replenish cDCs in other non-lymphoid tissues [11], [46], [57], but direct evidence for cDCs entering the dermis is still missing. Also, the migratory ability of moDCs to LNs is under debate. *De novo*-generated moDCs migrated from the inflamed dermis to LNs during *L. major* infection [16] but had limited migratory ability during contact hypersensitivity reactions [17]. We found that moDCs accumulated in the DENV-infected dermis and served as targets for virus replication, but migration of moDCs to draining LNs remains to be determined. In contrast, migration of LCs from the inflamed epidermis to LNs is well established [4]. Most LCs that exited the DENV-infected epidermis of AG129 mice [26] and human skin explants [25] were not infected. We found that LCs decreased in the epidermis (24 hpi) before they became DENV-infected by 48 hpi, and inoculation with PBS induced a similar decrease in LCs. Thus, inflammation rather than infection caused the decline in epidermal LCs. While LCs self-renew in the steady-state epidermis [4], recruitment of Ly6C^high^ monocytes replenishes LCs 4-7 days after irradiation with ultraviolet light [14]. We did not observe entry of Ly6C^high^ monocytes into the DENV-infected dermis up to 72 hpi, thus proliferation of resident LCs likely replenished LCs when frequencies recovered by 48 h.

Our findings in mice are consistent with DENV infection of primary human cells. *In vitro*-generated human moDCs were more susceptible to DENV infection than monocytes, cDCs, or macrophages under 1° infection conditions [23], [24], [58]. Here, we also find the highest DENV infection in moDCs, followed by cDCs, and less infection of monocytes and MΦs in the dermis. DC-SIGN (CD209) is an attachment factor for DENV, positively correlates with DENV infection under 1° conditions *in vitro*, and is highly expressed by human moDCs [58], [59]. *In vivo*, moDCs that accumulated in the LNs of mice during bacterial infection also expressed high levels of DC-SIGN [60], which likely explains the high susceptibility of moDCs to DENV infection that we find in the dermis. In contrast, human monocytes [22], [58] and MΦs [61] show some DENV infection under 1° conditions, but efficient DENV infection requires Fcγ receptor-mediated uptake of DENV-antibody complexes during ADE. Remarkably, Ly6C^high^ monocytes were recruited to the dermis by 12 hpi but became DENV-infected only 48-72 hpi under 1° or ADE conditions. Thus, local DENV titers, cell activation, expression of virus attachment factors, and/or the microenvironment likely influence DENV infection. Indeed, activation via the cytokines GM-CSF and IL-4 was necessary for DC-SIGN expression and DENV infection of human cDCs freshly isolated from the blood [23]. In our hands, ADE resulted in only a minor increase in DENV-infected moDCs in the skin, despite inducing severe disease and mortality in *Ifnar* ^–/–^ mice later on. This suggests that ADE acts mostly after systemic virus spread to increase infection and pathogenesis.

We further find that DENV infection blocked the expression of activation markers CD80 and CD86 on monocytes, DCs and MΦs in the dermis and that this effect was more pronounced during ADE compared to 1° infection. However, activation markers were upregulated in non-infected bystander cells within the DENV-infected dermis. These findings are in line with previous studies showing that non-infected bystander moDCs upregulate MHC I and II, as well as CD80, CD83, and CD86, in DENV-infected cultures [41], [42]. However, this activation was inhibited in DENV-infected moDCs within the same cultures, as revealed by intracellular staining for DENV proteins [41], [42]. Further studies are needed to determine the impact of impaired activation of DENV-infected DCs on the priming of DENV-specific naïve T cells and on maintaining effector T cell responses in the periphery.

Human cells infected with DENV become deficient in IFN-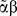 receptor signaling and production of IFN-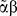 because DENV proteins NS5 and NS2B/3 degrade human STAT-2 [62] and STING [33], respectively. However, DENV proteins cannot bind and degrade the mouse homologs for STAT-2 [32] and STING [33]. We found here that i.d.-inoculated DENV did not replicate in the skin or cause disease in WT mice, similar to studies showing that intravenously infected WT mice did not show systemic DENV replication [31]. Previous models of DENV infection have used AG129 mice deficient in the IFN-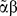 as well as IFN-γ receptor [29], [34], [63]-[66]. This model was improved recently by generating DENV strains, such as D220 used here, that efficiently replicate in less immunocompromised *Ifnar^–/–^* mice and cause disease with key features of dengue in humans [36], [67]. We found equal cell populations in the steady-state skin of WT and *Ifnar^–/–^* mice, and adoptive transfers of monocytes from WT and *Ifnar^–/–^* origin showed equivalent recruitment and differentiation during DENV infection. These results support that the current *Ifnar^–/–^* model is suitable to study early DENV infection and the recruitment of immune cells in the skin. Nevertheless, although suitable to study early DENV replication and recruitment of monocytes to the skin, the absence of IFNAR signaling may have effects on the subsequent priming of adaptive immune responses.

In summary, we demonstrate that dermal cDCs and MΦs are the initial targets for DENV infection at the site of transmission in the skin. Further, we reveal a new viral strategy exploiting monocyte recruitment and differentiation to moDCs to increase the number of targets for DENV replication in the dermis. These results should stimulate future studies on the role of dermal DC subsets in dengue pathogenesis and in priming protective immunity during vaccination or natural infection. Thus, these findings open possibilities for early DENV control, as the skin may be a site for therapeutic action or intradermal vaccination.

## Materials and Methods

### Ethics statement

Mice were bred and experiments were performed at the University of California Berkeley Animal Facilities strictly following guidelines of the American Veterinary Medical Association and the Guide for the Care and Use of Laboratory Animals of the National Institutes of Health. The Animal Care and Use Committee of the University of California Berkeley has approved all experiments (protocol R252-1012B). Trained laboratory personnel performed anesthesia of mice via isoflurane inhalation and euthanasia of mice using exposure to isoflurane followed by cervical dislocation.

### Mice

C57BL/6 wild-type (WT) mice were obtained from Jackson Laboratory, and C57BL/6 mice deficient in the IFN-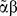 receptor-1 (*Ifnar^1tm1Agt^*, here called *Ifnar* ^–/–^) [68] were obtained from Dr. Daniel Portnoy (University of California Berkeley, Berkeley, USA). DENV2-infected animals were monitored using a morbidity scale as follows: 1, healthy; 2, mild signs of lethargy; 3, mild signs of lethargy, fur ruffling, hunched posture; 4, increased lethargy, limited mobility, ruffled fur, hunched posture; 5, moribund with minimal mobility and inability to reach food or water [36]. Moribund mice were euthanized immediately, scored as 5 and omitted from the mean morbidity on later days.

### Dengue virus

The study performed here examines the initial infection events in the skin; therefore, we used mosquito-derived virus, produced in *Aedes albopictus* C6/36 cells, that best mimics the natural cycle of DENV transmission from an infected mosquito to the mammalian host. Ten passages of the clinical DENV2 isolate PL046 between C6/36 cells and serum of 129/Sv mice deficient in IFN-α/β and -γ receptors generated the strain D2S10 [34]. Ten further passages of D2S10 by the same scheme resulted in the strain D220 [36] used throughout this study. Defined mutations that resulted from the passaging procedure have been identified [34], [36]. To grow DENV2 stocks, C6/36 cells (obtained from Paul R. Young, University of Queensland, Brisbane, Australia) were maintained at 28°C in M199 medium supplemented with 10% fetal bovine serum (FBS), 100 U/ml penicillin-streptomycin, 10 mM HEPES, and GlutaMAX (all obtained from LifeTech), rinsed with serum-free medium, and inoculated with DENV2 in RPMI 1640 medium containing 2% FBS. DENV2 was harvested on days 5 through 8 post-infection and concentrated with Amicon Ultra-15 Centrifugal Filter Units with 100 kDa molecular weight cut-off (Millipore). Virus titers were determined by plaque assay using BHK-21 clone 15 (BHK) cells maintained at 37°C and 5% CO_2_ in α-MEM medium supplemented with 5% FBS, 100 U/ml penicillin-streptomycin, and 10 mM HEPES. BHK cells were seeded in 12-well plates (Becton Dickinson), and at 60% confluence were inoculated with 150 µl of 10-fold serial dilutions of DENV2 stocks and incubated for 2 h before overlaying with 10% low-melting Seaplaque Agarose (Cambrex) in MEM medium. Plaques were counted 7 days later after fixation in 10% buffered formalin phosphate (Fisher Scientific), and titers were expressed as PFU/ml.

### Intradermal infections

WT and *Ifnar* ^–/–^ mice were inoculated i.d. with 10^6^ PFU DENV2 in 20 µl PBS using 30-gauge, 25-mm long, 10°–12° beveled removable needles and 25- µl glass syringes (Hamilton). For i.d. inoculations, ears of anesthetized mice were immobilized with cover slip forceps, and the needle was inserted parallel to the skin’s surface. DENV2-injected ears were compared to PBS-injected and steady state, untouched ears. Separate sets of needles and syringes were reserved for DENV and PBS injections and were cleaned by flushing with Lysol, sterile PBS, and 70% ethanol. For 1° infection conditions, naïve mice were used. For ADE infection conditions, 5 µg anti-DENV E monoclonal antibody 4G2 were injected in 200 µl PBS into the peritoneum 24 h prior to i.d. DENV infection.

### Sample preparation

Mice were euthanized 12, 24, 48, and 72 hpi, and organs were harvested. Ears were removed at the base, incubated for 5 min at room temperature with hair remover lotion (Nair), washed in D-PBS, and split into dorsal and ventral halves using fine-tip tweezers (TDI). Ear halves were digested with 2 U/ml Dispase II in HBSS with no Ca^++^/Mg^++^ (LifeTech) in 5% CO_2_ for 90 min at 37°C [69], while floating with the epidermal side up to achieve digestion through the dermal side [70]. The epidermis was removed as a sheet from the dermis, and both layers were cut into small pieces in separate 1.5 ml tubes using scissors. Epidermal and dermal layers were digested for 45 and 75 min, respectively, in 1.6 mg/ml collagenase type 1 (LifeTech) and 10 U/ml DNase 1 (Roche) in RPMI 1640 medium supplemented with 10% FBS at 37°C while shaking at 220 rotations per min. Homogeneous cell suspensions were generated via pipetting, and samples were filtered through 100 µm nylon meshes.

### Antibodies and staining

Staining with Zombie Aqua (BioLegend) or 7-AAD (eBiosciences) viability dyes excluded dead cells from general analysis. Specific staining of surface markers distinguished cell types using monoclonal antibodies from BioLegend, if not stated otherwise: CCR2 (clone 475301, R&D), CD11b (M1/70), CD11c (N418), CD45 (30-F11), CD80 (16-10A1), CD86 (PO3), CD103 (2E7), Ly6C (AL21), Ly6G (1A8), MHC II (I-A/I-E, M5/114.15.2), and Armenian hamster IgG (HTK888) or rat IgG2b isotype controls (RTK4530, BioLegend; or 141945, R&D), which were conjugated to PacificBlue, Brilliant Violet 605, PE, PE-CF594, PE-Cy7, Alexa Fluor 700, APC-Cy7, or biotin. Antibody stains were performed in D-PBS with no Ca^++^/Mg^++^ containing 2% FBS and 2 mM EDTA (LifeTech). Biotinylated antibodies were visualized using streptavidin conjugated to Brilliant Violet 605 or PE-Cy7 (BioLegend). After fixation with 2% formaldehyde (Ted Pella), cells were permeabilized with 1 mg/ml saponin solution (Sigma) containing 2% FBS and 1% normal mouse serum obtained from steady-state mice. Intracellular staining for DENV proteins E (4G2, ATCC) and NS3 (E1D8, [35]), which were conjugated to Alexa Fluor 488 or Alexa Fluor 647, respectively, using protein-labeling kits (LifeTech), identified DENV2-infected cells. Intracellular staining for Langerin (4C7) was used to further dissect cell populations.

### Flow cytometric analysis

Flow cytometry data were recorded with an LSR Fortessa cell analyzer (BD Biosciences) with 405, 488, 561, and 632 nm laser excitation lines and were analyzed using FlowJo 8.8.7 software (TreeStar). Gating FSC-A/SSC-A, SSC-H/SSC-W, FSC-H/FSC-W, and negative for Zombie Aqua or 7-AAD defined single, live-cell populations.

### Monocyte adoptive transfers

Total bone marrow cells were isolated from steady-state *Ifnar* ^–/–^ or WT mice by crushing femur, tibia and vertebral column with a mortar and pestle and filtering through nylon meshes. Centrifugation on Ficoll-Paque Plus (GE Healthcare) isolated bone marrow mononuclear cells, and incubation with 5% normal rat serum blocked unspecific binding to Fc receptors. The MACS Isolation Kit (Miltenyi Biotec) was then used to isolate monocytes via depletion of other cell types, without using the Fc receptor block provided. After incubation with the monocyte biotin-antibody cocktail and anti-biotin MicroBeads, monocytes were collected as run-through from LS columns in a magnetic field. Monocytes were labeled for 8 min at 37°C with 2 µM CFSE (Invitrogen) in PBS with 1% FBS and washed twice in ice-cold PBS with 10% FBS before adoptive transfer.

### Statistical analysis

Kaplan-Meier curves display survival data, and statistical significance between experimental groups was determined using the Log-rank (Mantel-Cox) test. The Mann-Whitney unpaired, non-parametric test was used to determine significant differences between experimental groups of data pooled from independently repeated experiments, depicted in Figures as mean ±SEM, if not stated differently. *P*-values were considered significant at values ≤0.05; and *p*-values summarized on graphs are shown as non-significant (NS) for *p*>0.05; * for *p*≤0.05; **, *p*≤0.01; ***, *p*≤0.001; and ****, *p*≤0.0001. Data were plotted and statistically analyzed using Prism 6.0 software (GraphPad).

## Supporting Information

Figure S1 **Related to** **Fig. 1:** **No disease in WT mice after i.d. inoculation with DENV2.** (A and B) WT mice were injected i.d. with 10^6^ PFU DENV2 under 1° (A) or ADE (B) infection conditions. Mean morbidity + SEM on a scale from 1 =  healthy to 5 =  moribund. The dotted line marks the time-point of 72 h, when symptoms of disease first appeared. (C) Survival of i.d. DENV2-infected WT mice (data pooled from two independent experiments, *n* = 6–7 per condition).
(TIF)

Figure S2 **Related to** **Fig. 2:** **Phenotype of DENV-infected cells in the epidermis under ADE infection conditions.** MHC II and Langerin expression of all CD45^+^ cells that were gated DENV NS3^+^ E^+^ 72 h after i.d. inoculation with DENV2 under ADE conditions. Representative plots are from 3 independent experiments (*n* = 10).
(TIF)

Figure S3 **Related to** **Fig. 3:** **Phenotype of MΦs.** (A) F4/80 and MHC II expression of MΦs in the dermis of steady-state WT and *Ifnar* ^–/–^ mice, gated as MHC II^low/–^ CD11b^+^ Ly6G^–^ Ly6C^low/–^. (B) FSC/SSC profile of MΦs compared to Ly6C^high^ monocytes. Representative data from two independent experiments (n = 7 per mouse strain).
(TIF)

Figure S4 **Related to** **Fig. 4:** **Cell populations in the DENV-infected dermis under ADE conditions or after inoculation with PBS.** (A and B) *Ifnar* ^–/–^ mice were inoculated with DENV2 under ADE infection conditions (A) or with PBS (B). The dermis was harvested and dermal cell populations were analyzed after 48 h. Representative plots of four independent experiments are shown (*n* = 10 per condition).
(TIF)

Figure S5 **Related to** **Fig. 8:** **Phenotype and purity of monocytes isolated from the bone marrow and their engraftment into the steady-state dermis.** (A) Staining and gating of isolated monocytes or all bone marrow mononuclear cells. The purity of CD11c^–^ MHC II^–^ Ly6G^–^ CD11b^+^ monocytes isolated from *Ifnar* ^–/–^ donors was 91% (A) and from WT donors was 95%, while bone marrow mononuclear cells contained 19% and 21% monocytes, respectively. Further, surface expression of CCR2 compared to isotype-matched controls of isolated Ly6C^high^ monocytes are depicted. (B) Isolated *Ifnar* ^–/–^ monocytes were labeled with CFSE, and 9×10^6^ cells were transferred intravenously into 4 week-old steady-state *Ifnar* ^–/–^ recipients. Contour blots show CD45^+^ cells and gating of CFSE^+^ graft and CFSE^–^ host cells in the steady-state dermis, 72 h after transfer of monocytes. CFSE^+^ graft and CFSE^–^ host cells were gated CD11b^+^ Ly6G^–^ and MHC II^–^ Ly6C^+^ for monocytes or MHC II^+^ Ly6C^+^ for moDCs, as indicated. Representative plots of two independent experiments (*n* = 4 steady-state recipients or *n* = 2 steady-state non-transplanted controls).
(TIF)
