# Supplementary figures and images for: Monocyte Recruitment to the Dermis and Differentiation to Dendritic Cells Increases the Targets for Dengue Virus Replication
Source: PLoS Pathog. 2014 Dec 4;10(12):e1004541. doi: 10.1371/journal.ppat.1004541 (PMC4256458; doi:10.1371/journal.ppat.1004541)

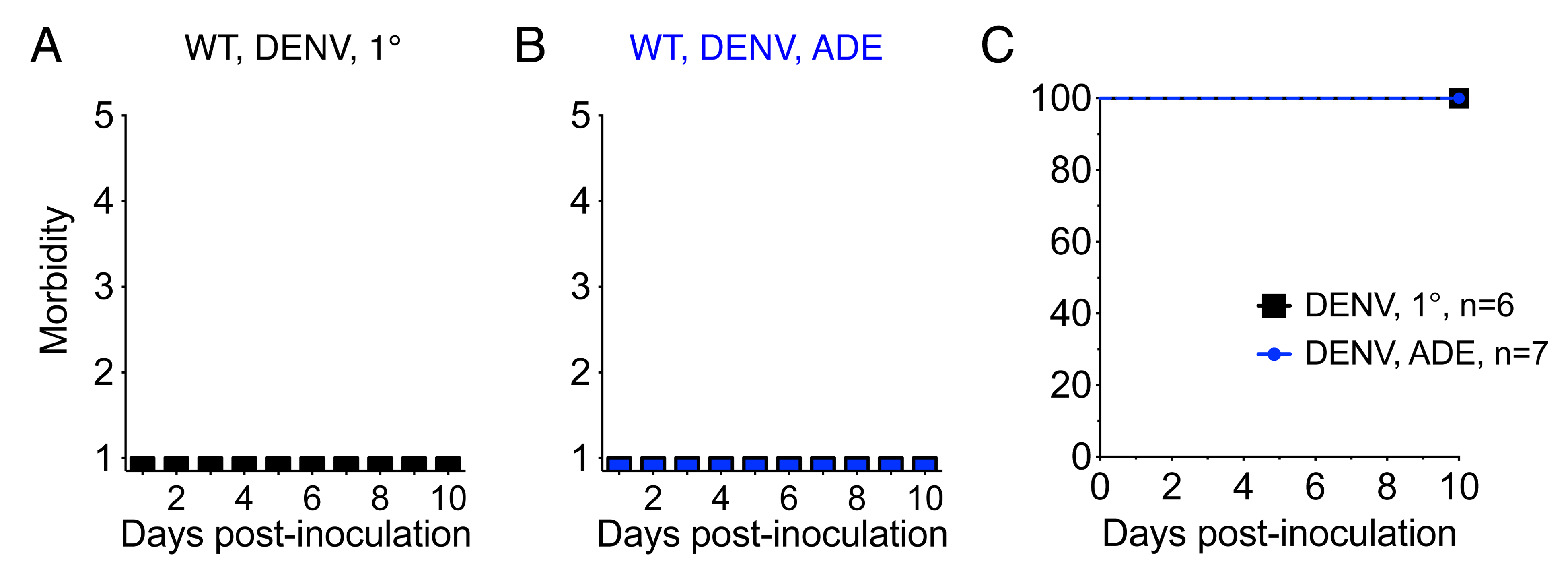

Supplement: Figure S1 — Related to Fig. 1: No disease in WT mice after i.d. inoculation with DENV2. (A and B) WT mice were injected i.d. with 106 PFU DENV2 under 1° (A) or ADE (B) infection conditions. Mean morbidity + SEM on a scale from 1 = healthy to 5 = moribund. The dotted line marks the time-point of 72 h, when symptoms of disease first appeared. (C) Survival of i.d. DENV2-infected WT mice (data pooled from two independent experiments, n = 6–7 per condition). (TIF) [file ppat.1004541.s001.tif]

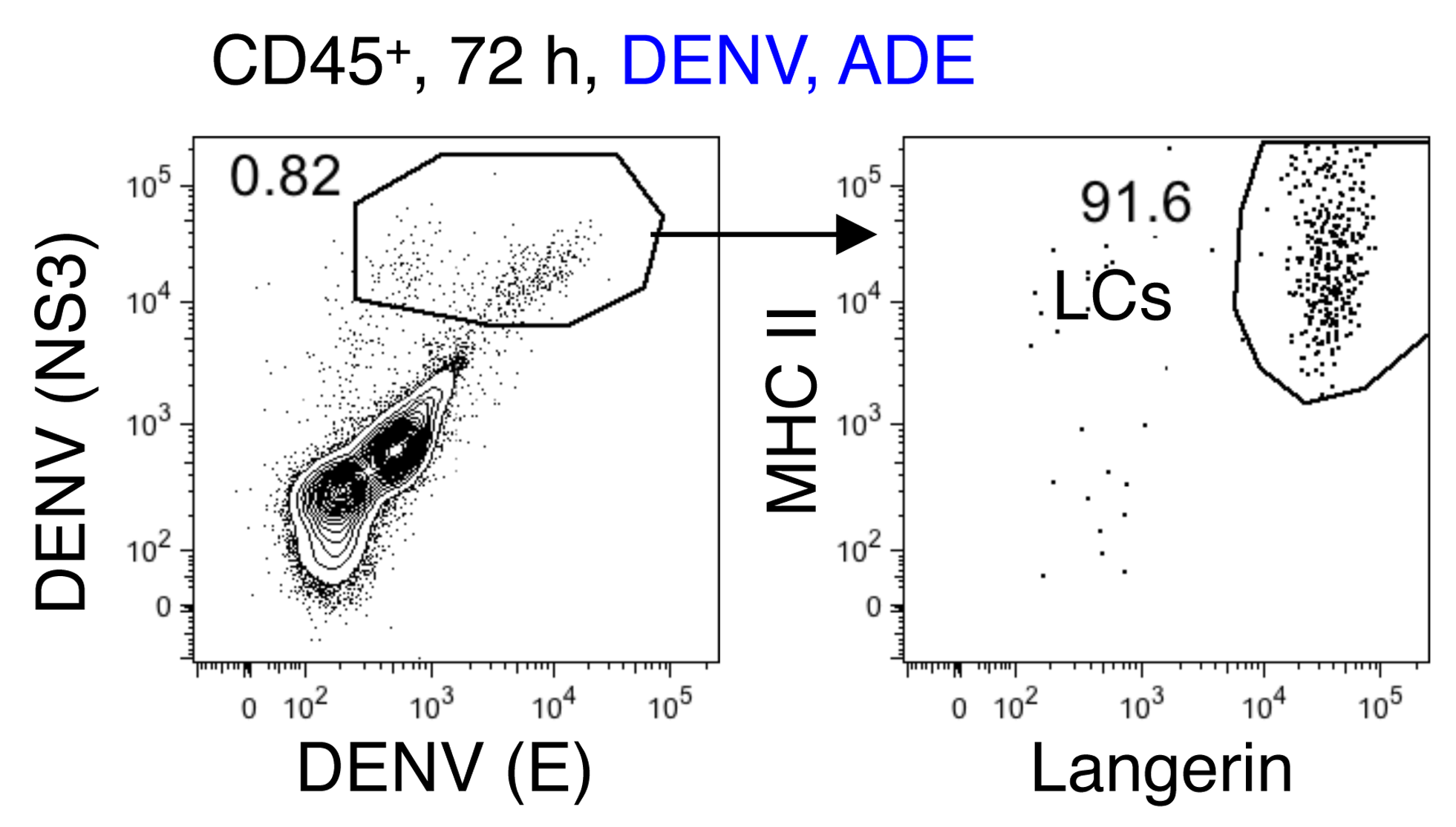

Supplement: Figure S2 — Related to Fig. 2: Phenotype of DENV-infected cells in the epidermis under ADE infection conditions. MHC II and Langerin expression of all CD45+ cells that were gated DENV NS3+ E+ 72 h after i.d. inoculation with DENV2 under ADE conditions. Representative plots are from 3 independent experiments (n = 10). (TIF) [file ppat.1004541.s002.tif]

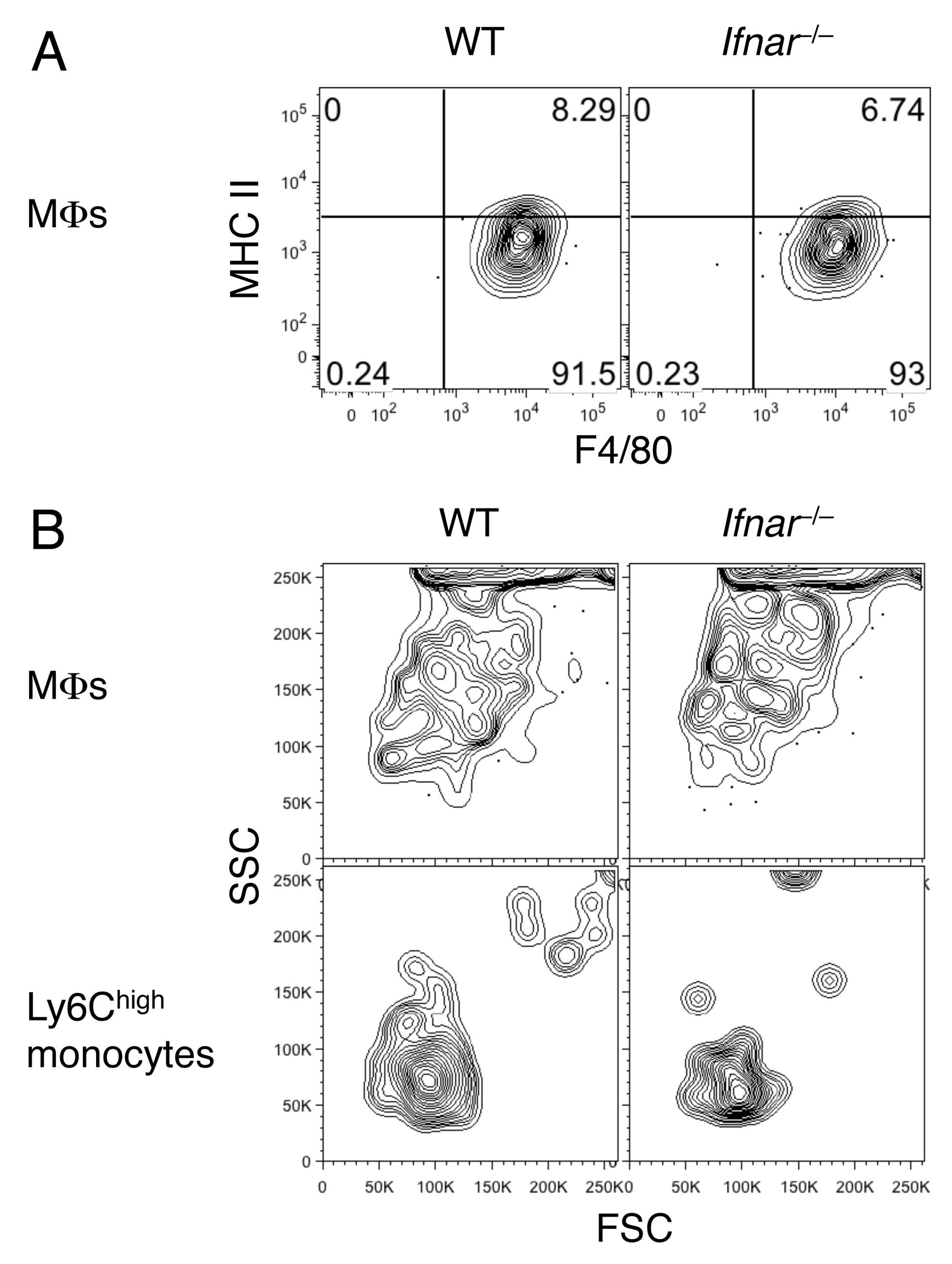

Supplement: Figure S3 — Related to Fig. 3: Phenotype of MΦs. (A) F4/80 and MHC II expression of MΦs in the dermis of steady-state WT and Ifnar –/– mice, gated as MHC IIlow/– CD11b+ Ly6G– Ly6Clow/–. (B) FSC/SSC profile of MΦs compared to Ly6Chigh monocytes. Representative data from two independent experiments (n = 7 per mouse strain). (TIF) [file ppat.1004541.s003.tif]

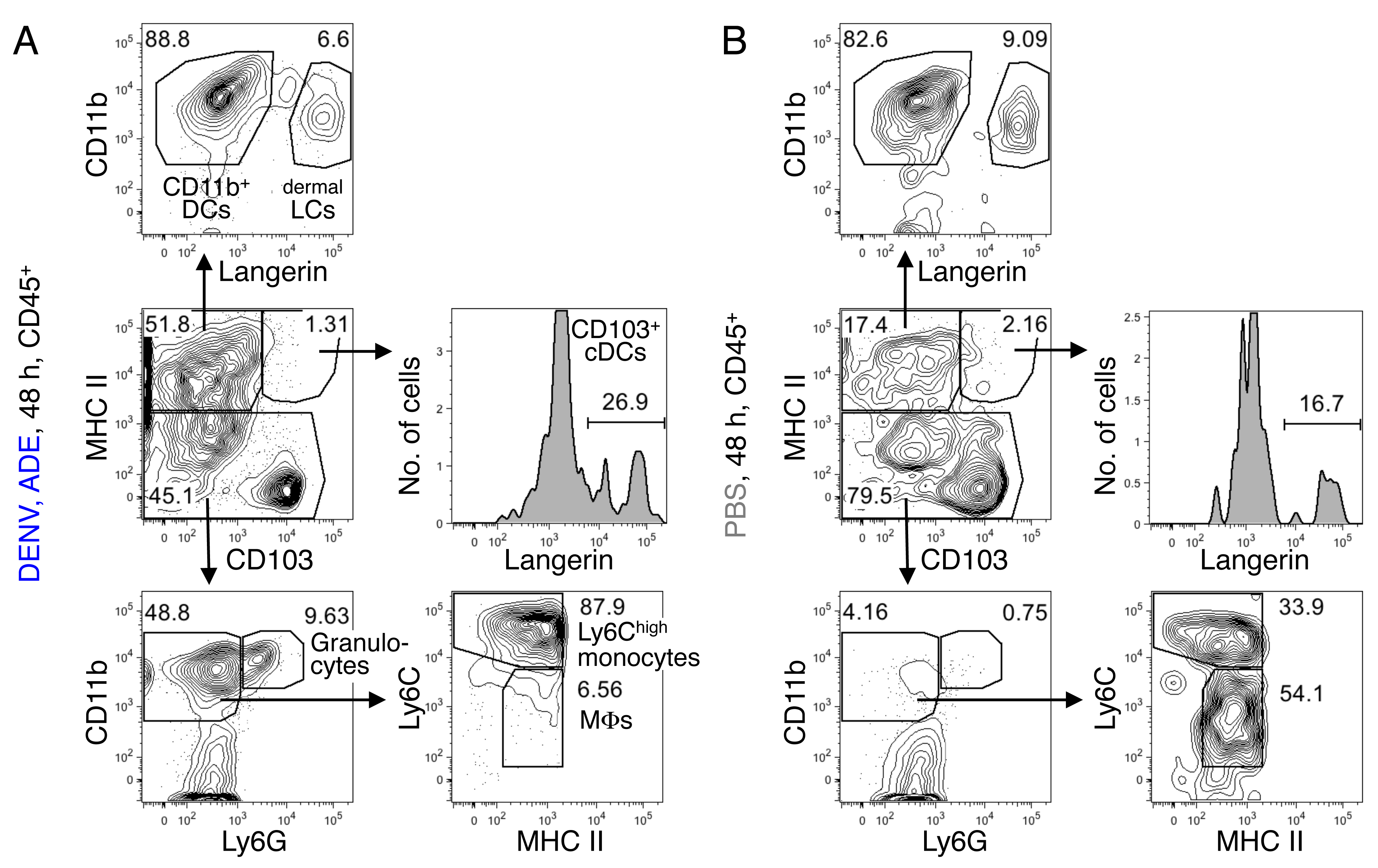

Supplement: Figure S4 — Related to Fig. 4: Cell populations in the DENV-infected dermis under ADE conditions or after inoculation with PBS. (A and B) Ifnar –/– mice were inoculated with DENV2 under ADE infection conditions (A) or with PBS (B). The dermis was harvested and dermal cell populations were analyzed after 48 h. Representative plots of four independent experiments are shown (n = 10 per condition). (TIF) [file ppat.1004541.s004.tif]

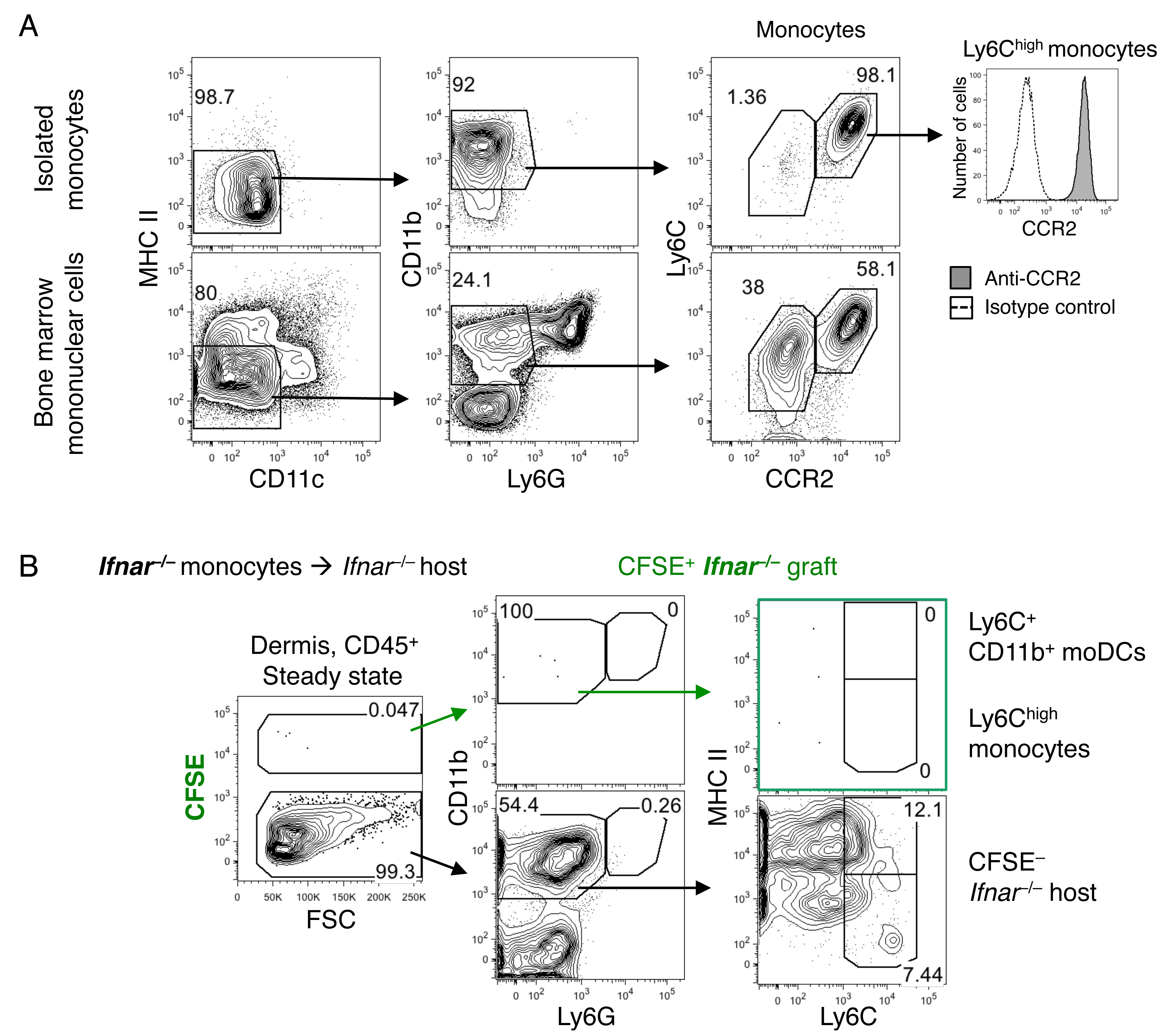

Supplement: Figure S5 — Related to Fig. 8: Phenotype and purity of monocytes isolated from the bone marrow and their engraftment into the steady-state dermis. (A) Staining and gating of isolated monocytes or all bone marrow mononuclear cells. The purity of CD11c– MHC II– Ly6G– CD11b+ monocytes isolated from Ifnar –/– donors was 91% (A) and from WT donors was 95%, while bone marrow mononuclear cells contained 19% and 21% monocytes, respectively. Further, surface expression of CCR2 compared to isotype-matched controls of isolated Ly6Chigh monocytes are depicted. (B) Isolated Ifnar –/– monocytes were labeled with CFSE, and 9×10^6^ cells were transferred intravenously into 4 week-old steady-state Ifnar –/– recipients. Contour blots show CD45+ cells and gating of CFSE+ graft and CFSE– host cells in the steady-state dermis, 72 h after transfer of monocytes. CFSE+ graft and CFSE– host cells were gated CD11b+ Ly6G– and MHC II– Ly6C+ for monocytes or MHC II+ Ly6C+ for moDCs, as indicated. Representative plots of two independent experiments (n = 4 steady-state recipients or n = 2 steady-state non-transplanted controls). (TIF) [file ppat.1004541.s005.tif]
